# Extracellular Superoxide Dismutase Regulates Early Vascular Hyaluronan Remodeling in Hypoxic Pulmonary Hypertension

**DOI:** 10.1038/s41598-019-57147-7

**Published:** 2020-01-14

**Authors:** Victor Tseng, Kevin Ni, Ayed Allawzi, Clare Prohaska, Laura Hernandez-Lagunas, Hanan Elajaili, Valbona Cali, Ronald Midura, Vincent Hascall, Barbara Triggs-Raine, Irina Petrache, C. Michael Hart, Eva Nozik-Grayck

**Affiliations:** 1 Emory University Department of Medicine, Atlanta, GA USA; 2 Indiana University School of Medicine, Indianapolis, IN USA; 3 Department of Pediatrics, University of Colorado Anschutz Medical Campus, Aurora, CO USA; 4 Cardiovascular and Pulmonary Research, University of Colorado Anschutz Medical Campus, Aurora, CO USA; 5 Department of Medicine, National Jewish Health, Denver, CO USA; 6 Department of Biochemistry and Medical Genetics, University of Manitoba, Winnipeg, MB Canada; 7 Lerner Research Institute, Cleveland Clinic, Cleveland, OH USA

**Keywords:** Mechanisms of disease, Glycobiology, Vascular diseases, Cardiovascular biology, Cardiovascular diseases

## Abstract

Chronic hypoxia leads to pathologic remodeling of the pulmonary vasculature and pulmonary hypertension (PH). The antioxidant enzyme extracellular superoxide dismutase (SOD3) protects against hypoxia-induced PH. Hyaluronan (HA), a ubiquitous glycosaminoglycan of the lung extracellular matrix, is rapidly recycled at sites of vessel injury and repair. We investigated the hypothesis that SOD3 preserves HA homeostasis by inhibiting oxidative and enzymatic hyaluronidase-mediated HA breakdown. In SOD3-deficient mice, hypoxia increased lung hyaluronidase expression and activity, hyaluronan fragmentation, and effacement of HA from the vessel wall of small pulmonary arteries. Hyaluronan fragmentation corresponded to hypoxic induction of the cell surface hyaluronidase-2 (Hyal2), which was localized in the vascular media. Human pulmonary artery smooth muscle cells (HPASMCs) demonstrated hypoxic induction of Hyal2 and SOD-suppressible hyaluronidase activity, congruent to our observations *in vivo*. Fragmentation of homeostatic high molecular weight HA promoted HPASMC proliferation *in vitro*, whereas pharmacologic inhibition of hyaluronidase activity prevented hypoxia- and oxidant-induced proliferation. Hypoxia initiates SOD3-dependent alterations in the structure and regulation of hyaluronan in the pulmonary vascular extracellular matrix. These changes occurred soon after hypoxia exposure, prior to appearance of PH, and may contribute to the early pathogenesis of this disease.

## Introduction

Pulmonary hypertension (PH) is an incurable and fatal disease characterized by intimal thickening, muscular hypertrophy, excessive matrix deposition, and stiffening of arteries in the lung. Chronic hypoxia is a major cause and consequence of PH in humans. It is the most prevalent etiology of precapillary PH in humans (WHO Group 3 disease). The onset of PH in patients with hypoxia due to intrinsic lung disease portends a grim prognosis for survival, post-transplant outcome, and quality of life^1,2^. Therefore, there is an urgent need for research on the molecular pathogenesis of hypoxic pulmonary hypertension.

One facet of this disease is the presence of high oxidative stress within the vessel wall and perivascular matrix^3^. Extracellular superoxide dismutase (SOD3) is crucial for neutralization of this stress. Global knockout^4^, selective deletion from smooth muscle cells^5^, and disrupted matrix-binding single nucleotide polymorphisms^6^ of SOD3 result in a great susceptibility to hypoxia-induced vascular inflammation, fibrosis, and PH. On the other hand, lung-specific overexpression of SOD3^7^, adenoviral gene transfer of human SOD3^8^, and treatment with an inorganic metalloporphyrin SOD3 mimetic^9^ are protective against PH in rodent models. In the advanced stages of PH in humans, SOD3 levels are epigenetically reduced by histone deacetylation^10^.

There is a critical gap in our understanding of how the extracellular oxidative environment can lead to irreversible pulmonary vascular remodeling in chronic hypoxia. Amassing evidence points to a central role for degradation of ECM molecules due to hypoxia-induced oxidant stress. Of these, hyaluronan (HA) is noteworthy due to its unique vulnerability to depolymerization through direct oxidation, acid hydrolysis, or enzymatic processes^11,12^. In healthy tissues, HA exists as a linear megadalton chain of ≥1000 repeating disaccharides of glucoronate and N-acetyl-glucosamine linked by a glycosidic bond. HA is the most rapidly catabolized and recycled glycosaminoglycan of the lung extracellular matrix. Cleavage of high molecular weight HA (HMWHA) into lower molecular weight (LMWHA) fragments and oligosaccharides (oligoHA) can significantly alter its signaling function. In diverse vascular pathologies, conversion of HMWHW to LMW-oligoHA is associated with inflammatory cell recruitment and activation^13,14^, increased endothelial permeability^15^, enhanced migration and proliferation of vascular smooth muscle cells^16^, and collagenous matrix secretion from adventitial fibroblasts^17^. Finally, the content of HA affects the vessel biomechanical properties such as tissue stiffness, hydration, and oncotic pressure^18,19^. Thus, HA is poised to serve as an important sensor and transducer of the extracellular redox environment around pulmonary vessels.

Abnormal HA production and breakdown has been identified in multiple preclinical models of PH and explanted human lungs^20,21^. In a monocrotaline model of rat PH, fragmentation of HA occurs during disease progression^22^ and is attributable to upregulation of hyaluronidase activity. Lungs of patients suffering from idiopathic PH (IPAH) show exuberant perivascular expansion of HA within plexiform lesions. Additionally, great quantities of HA are deposited around the diseased arteries in PH evolving from chronic obstructive pulmonary disease (COPD-PH)^23^ and idiopathic pulmonary fibrosis (IPF-PH)^24^.

To understand the regulation of HA fragmentation in the lung, we studied how SOD3 protects against HA breakdown in a mouse model of chronic hypoxia. SOD3 is intimately associated with matrix proteins such as collagen I, syndecan, and elastin; and proteoglycans including heparan sulfate (HS) and HA by ionic interactions through its C-terminal matrix binding domain. Thus, in SOD3-deficient states, these binding partners would be expected to bear the greatest impact from oxidant stress. Indeed, SOD3 is required to prevent HA fragmentation by ROS *in* vitro^25^, and protects against lysis and shedding of HA and HS side chains into the airspaces in an inhalational asbestos model of acute lung injury and fibrosis^26,27^. SOD3 also protected against cigarette smoke-induced pulmonary emphysema by suppressing the fragmentation of HS and elastin^28^. In contrast, chronic hypoxia is a more muted process, with a lesser rate of oxidative stress accumulation and inflammation compared to these chemically-induced lung injuries. Whether chronic hypoxia can provoke HA degradation, and if this is curtailed by SOD3, are therefore important open questions.

We hypothesized that SOD3 prevents the oxidative and hyaluronidase-mediated cleavage of HA, and is indispensable to maintain its integrity during hypoxia. We utilized mouse strains deficient in SOD3, exposed to chronic hypoxia, followed by quantitative, structural, and histologic characterization of lung HA.

## Methods

### Mice

8–10 week old male and female C57/B6 mice were used for all experiments. All animal protocols were approved by the Institutional Animal Care and Use Committee (IACUC) at the University of Colorado Anschutz Medical Campus, according to the guidelines established by international Association for Assessment and Accreditation of Laboratory Animal Care (AAALAC). Mice were housed with a standard 12-hour light/dark cycle and fed standard chow and water *ad libitum*. The SOD3^−/−^ (SOD3KO) mouse colony^29^, originally a gift from James Crapo (National Jewish Health), was maintained in-house and is also available from Jackson Laboratories. ER_2_-SMMHC-SOD3^loxp^ (SMC-SOD3Flox) and ER_2_-SMMHC-Cre/SOD^loxp^ (SMC-SOD3cKO) were generated by Dr. David Harrison^5^ (Emory University, Atlanta, GA). Induction of Cre recombinase for SOD3 knockout was accomplished by intraperitoneal (IP) injection of 20 mcg/gm body weight tamoxifen dissolved in sterile corn oil on five consecutive days. SMC-specific SOD3 knockout was confirmed by western blotting for SOD3 in abdominal aortas. Following assessment of PH, mice were euthanized by isoflurane overdose followed by cervical dislocation and bilateral thoracotomy. Lungs were lavaged with 5 mL of PBS and subsequently perfused through the RV until blanched. Whole blood was collected in non-heparinized tubes to retrieve serum.

### Animal hypoxia model

Mice were exposed to chronic hypobaric hypoxia for 14 and 35 days. Briefly, animals were placed in hypobaric chambers evacuated to a barometric pressure of 395 mmHg, simulating an equivalent inspired O_2_ fraction of 10%. Atmospheric pressures and CO_2_ concentrations were monitored continuously.

### Assessment of pulmonary hypertension

Mice were sedated with 1.5% isoflurane under a nosecone. Right ventricular systolic pressure (RVSP) was determined by subxyphoid closed chest puncture with a 24-gauge needle attached to a digital pressure transducer. A 30-second interval comprising eligible parameters for HR (400–500 bpm), end-diastolic pressure (≤5 mmHg), and systolic variation (<10%) were selected for analysis (Cardiomax 3, Columbus Instruments; Columbus, OH). RVSP measurements were excluded if readings fell outside of these limits (excluded: 4 WT and 5 SOD3KO mice). RVH was reported by Fulton’s Index of RV/(LV + Septum) mass (excluded: 3 WT and 1 SOD3KO hearts due to allocation for histologic analysis). RV contractility was determined in a separate cohort of mice by calculating the maximal instantaneous rate of pressure rise (+dP/dt_*max*_) during systole. Contractility remained >1000 mmHg/sec in all mice at 14 and 35 days of hypoxia, indicating preserved RV function (data not shown).

### Determination of HA content

HA concentrations were assayed using the Aggrecan HA-binding protein (HABP) G1 Link-domain based ELISA-like assay (Quantikine, R&D Systems; Minneapolis, MN). Lungs were completely perfused and lavaged to remove the alveolar pool of HA. Total lung homogenates were prepared in T-PER protein extraction buffer (Thermo-Fisher; Waltham, MA) with protease and phosphatase inhibitors added at 1:100 (Sigma-Aldrich; St. Louis, MO). Total protein was quantitated using the Pierce BCA method. The ELISA was loaded with 1 µg lung protein, serum diluted to 1:80 or BAL fluid diluted 1:2 in PBS, and the assay was performed exactly according to the manufacturer protocol. Optical density was taken at 450 nm (colorimetric signal) and 540 nm (background) in standard plate reader (Omega, BMG LabTech; Ortenberg, Germany). Sample concentrations were fit to the standard curve generated by 4-parameter logistic (4PL) regression (ELISAKit software, Melbourne, Australia). Samples with OD exceeding the upper limit of linearity were diluted and repeated.

### Fluorophore-assisted carbohydrate electrophoresis (FACE)

A potential limitation of the HABP ELISA-like assay is the lower sensitivity for detection of very low molecular weight (LMWHA) and oligomeric fragments of HA^30^. We therefore performed FACE analysis to confirm ELISA results. FACE is a well-validated method to obtain highly accurate picomolar quantitation of glycan species by reductive amination^31^. Briefly, a 1 µL aliquot of the resuspended glycosaminoglycan pellet (from sample preparation for fragmentation analysis, described below) was digested with excess *Streptococcus dysgalactiae* hyaluronidase to cleave HA into unit disaccharides. The solution of disaccharides was then lyophilized. The dissacharides were reductively aminated by adding 1 µL/2.5 mg wet tissue of 2-aminoacridone (AMAC, 6.25 mM) in 1:1 mixture with 2 M sodium cyanoborohydride, followed by incubation for 18 hours at 37 °C. The samples were then loaded onto a 40% acrylamide gel and vertically electrophoresed (TetraCell, Bio-Rad) at 5 °C for 1 hour at 500 V. FACE standards, with known concentrations of HA *n*-mers, were used for calibration. The gels were then transferred to a UV imager for fluorescence quantitation. Absolute HA concentrations were determined by normalization against the HA-2 disaccharide standard, and then against dsDNA loaded as described below.

### HA fragmentation and size dispersity analysis

We adapted the protocol used by the Cleveland Clinic Programs of Excellence in Glycosciences^32^. Frozen lung samples (100 mg) were proteolyzed in 10X proteinase K dissolved in 100 mM ammonium acetate (1 mg/mL) with 0.01% SDS. Proteinase K was heat-inactivated at 100 °C for 5 minutes. The suspension was centrifuged at 14,000 g for 10 minutes. Lipids were extracted and glycosaminoglycans were precipitated overnight in chilled (−20 °C) 200 proof ethanol. The resulting glycosaminoglycan-containing pellet was washed with 75% ethanol and resuspended in 0.1 M ammonium acetate. For loading normalization, the concentration of dsDNA was determined using PicoGreen plate assay (Molecular Probes; Eugene, OR) and microvolume spectroscopy (NanoDrop One; Thermo-Fisher). Nucleic acids were then digested overnight at 37 °C with Benzonase 250 U/µL at 1:20 by volume (EMD Millipore; Burlington, MA). The Benzonase was heat-inactivated at 95 °C for 5 min. A second extraction in cold ethanol overnight to enrich GAGs within the pellet was performed as before. The samples were then divided, with half being digested with 0.2 turbidity reducing units (TRU) *Streptomyces* hyaluronidase (Seikagaku Co; Tokyo, Japan) overnight at 37 °C followed by heat-inactivation at 95 °C for 5 min. The undigested and hyaluronidase-digested (negative control) samples were lyophilized on a heated vacuum centrifuge at >2000 rpm and 65 °C for 2 hours. The remaining purified glycans were resuspended in 10 M formamide. A 1% high optical clarity agarose gel was casted (Seakem HGT, Lonza; Basel, Switzerland) and pre-run for 6 hours. The samples were electrophoresed on the gel at 100 V × 2 hours in TAE buffer. HA standards comprising a ladder between 2000 kD and 50 kD were provided at 7 µg/µL by the Cleveland Clinic. The gel was then equilibrated for 1 hour in 30% ethanol and stained overnight in a 1:400 solution of Stains-All (Sigma). The following day, the gel was equilibrated in water and briefly destained by exposure to ambient light. HA was imaged by UV epifluorescence using the Cy5-695/55 filter on a ChemiDoc MP gel imager (image acquisition protocol for intermediate and low MW HA) or standard color scanner (protocol for HMWHA). Densitometry was performed in ImageJ.

### Detection of HA heavy chain (HC) modification

We utilized the protocol developed by Mark Lauer *et al*.^33^. Frozen lung samples (50 mg) were homogenized in PBS for 90 seconds using 1.6 mm ceramic beads (OMNI, Kennesaw, GA). The samples were then rested at 4 °C for 30 minutes and divided into equal portions. PBS (control) or 0.2 TRU hyaluronidase was added, followed by incubation at 37 °C for 45 minutes. The samples were denatured and reduced in Laemmli buffer (NuPage LDS with reducing agent). Digested and undigested (control) samples were loaded side by side and electrophoresed at 200 V on a 7.5% stain-free PAGE gel (Criterion TGX; BioRad; Hercules, CA). Total protein loading was determined by UV activation of the stain-free gel for 1 minute and imaging on a ChemiDoc MP. Protein was then blotted to a PVDF membrane with a vertical field transfer system (Turbo-Blot; BioRad) using a 0–45 V gradient applied over 45 minutes. The membrane was blocked in 5% milk/TBST and then vigorously washed. Rabbit-anti-Mouse Inter-α-Inhibitor (IαI) antibody (DAKO, A0301) was used to probe the membrane at 1:1000 dilution overnight at 4 °C. The blot was then washed and 2° antibody (Goat-anti-Rabbit IgG) was applied at 1:10,000 in 5% milk/TBST. Membranes were then washed and developed with peroxidase enhanced chemiluminescence (ECL) reagent (Clarity ECL, BioRad) for 1 minute and imaged on a ChemiDoc MP. HC-HA complexes are impermeable to the gel, and thus HC was identified as a band alternately present (hyaluronidase-digested) and absent (undigested) at 74kD according to previously published protocols validating the antibody in mouse lungs^33^. Densitometry was performed on ImageJ and normalized to total protein.

### Hyaluronan degrading activity

For semi-quantitative visualization of total lung HA-degrading ability, protein (40 µg) obtained from whole lung lysates was diluted in PBS and incubated with 7.5 µg of HMWHA (1 MDa) or 3 µg IMWHA (500 kDa Select-HA, Hyalose; Millipore Sigma) at 37 °C for 50 minutes. To demonstrate the hyaluronidase-dependent nature of HA degradation, selected lysates from 7d hypoxia SOD3KO group were preheated to 100 °C for 30 minutes or proteolyzed with Proteinase K (1 mg/mL with SDS at 0.01%) for 3 hours at 55 °C to inactivate hyaluronidases. *Streptomyces* hyaluronidase (4 × 10^−4^) was added to the heat and protease-killed lysates as a positive control. To isolate the contributions of superoxide-induced versus enzymatic cleavage of HA, protein lysates were incubated with 100 units of bovine erythrocyte SOD1 (Sigma) versus PBS for 15 minutes at room temperature prior to addition of HA. The samples were then heat-killed at 90 °C for 5 minutes. Gel electrophoresis and HA staining were carried out as described above. Total HA degradation was quantitated as the ratio between digested and undigested HA, and by a mobility shift in the center of mass of the densitometry distribution along the Y axis. The center of mass along the y-(vertical) axis was determined using the Y_M_ function in ImageJ. This is calculated as ${{\rm{Y}}}_{{\rm{M}}}=\sum _{p}({{\rm{I}}}_{p}\cdot {{\rm{Y}}}_{p})/{{\rm{I}}}_{total}$ where I_*p*_ is the intensity of across all horizontal pixels at vertical coordinate Y_*p*_, and I_*total*_ is the total intensity of all pixels in the region analyzed.

### RT-qPCR for HA-regulating gene expression

Frozen lungs were pulverized with a mortar and then homogenized on ice for 30 seconds with a handheld high speed rotor. RNA was obtained by addition of Cotrimoxazole lysis reagent (QIAzole, QIAGEN; Hilden, Germany) followed by extraction with chloroform added in a 1:5 ratio. Following precipitation with 100% ethanol, samples were passed through an RNA-binding spin column (miRNeasy QIAGEN). On-column digestion was performed with 30 Kunitz units of DNase-I for 15 minutes. The column was washed and mRNA eluted with water. Total RNA was quantified by A260/280 ratio via NanoDrop. cDNA synthesis was performed by reverse transcription reaction (iScript, BioRad) using 2000 ng of template. For qPCR reactions, between 20–40 ng of cDNA were utilized per well with TaqMan Fast Advanced Master Mix. No-template controls were included in each experiment. TaqMan probes (Thermo-Fisher) for relevant murine HA-related genes were as follows: *has2* (Mm00515089_m1), *hyal2* (Mm01230688_g1), *hyal1* (Mm00476206_m1), *kiaa1199/cemip* (Mm00472921_m1), *tmem2* (Mm00459599_m1), *tsg6* (Mm00493736_m1). PCR in human PASMCs Hyal2 was was performed with the *hyal2* (Hs00186841_m1) probe. Relative expression (Rq) against the housekeeping gene *hprt* (Mm03024075_m1) for mouse samples, or 18 S ribosomal RNA (Hs03003631_g1) in HPASMCs was determined via the double delta C_t_ method. Fluorometric amplification signal was monitored in real time (QuantStudio 7 Flex thermocycler, Applied Biosystems; Foster City, CA). Replicates were included only if the standard deviation of C_t_ values was <0.5. If housekeeping C_t_ values were within ±1 cycle, Rq values from multiple experiments were pooled after normalization to wild-type normoxic group mean.

### Protein immunoblotting

Protein was isolated from lung tissue in T-PER buffer and quantified with BCA assay as described above. Protein from cultured cells was isolated in RIPA lysis buffer (Thermo-Fisher). Under reducing conditions, protein was loaded onto a 4–12% precast gradient PAGE gel (Criterion Bis-Tris HCl XT, BioRad). Samples were electrophoresed at 200 V for 55 minutes. The protein was then transferred onto PVDF as described before. The membrane was blocked in 5% milk/TBST for 1 hour and probed with rabbit anti-mouse polyclonal HYAL2 antibody diluted to 1:500 (Abcam #68608; Cambridge, UK) overnight at 4 °C. This antibody has been used to detect HYAL2 in human aortic valve interstitial cells cultured in hypoxia^34^. SOD3 from HPASMCs was probed with mouse-anti-human monoclonal antibody (sc-376948, Santa Cruz Biotech; Dallas, TX). Loading controls were either mouse monoclonal β-actin (Sigma #A2228, used at 1:10 K) or rabbit-anti-human polyclonal Vinculin (Cell Signaling Technologies #4650, used at 1:1 K). The membranes were then probed with mouse polyclonal IgG-HRP 2° antibody (EMD Millipore #AP124P, used at 1:10 K) or goat anti-rabbit 2° antibody at 1:10 K for 1 hour. Blots were developed with Clarity ECL, imaged under trans-UV, and quantitated by relative densitometry as previously described.

### Immunohistochemistry

Lungs were inflation-fixed with 4% paraformaldehyde (PFA) at 20 cmH_2_O column pressure. After equilibration in PFA for 24 hours, they were transferred to 70% ethanol and embedded in paraffin. The lung tissue blocks were cut to 5 micron thickness. The slides were deparaffinized with Citrasolv for 10 minutes. They were then sequentially rehydrated in baths of 100%, 90%, and 70% ethanol followed by PBS. Antigen retrieval was carried out with 10 mM sodium citrate (pH 6) in a pressure cooker set on high for 5 minutes. After cooling, the slides were rinsed with PBS and quenched in 0.3% hydrogen peroxide for 30 minutes.

#### Hyal2 staining

The slides were blocked in 10% horse serum for 1 hour. Rabbit polyclonal anti-Hyal2 primary antibody (Abcam #68608) was added at 1:120 dilution in 1% horse serum with PBS and incubated overnight at 4 °C. The next day, universal mouse anti-rabbit IgG HRP 2° antibody (ImmPRESS Reagent RTU; VectorLabs; Burlingame, CA) was added and incubated for 30 minutes.

#### Co-staining of α-smooth muscle actin (α-SMA) and hyaluronan binding protein (HABP)

The slides were blocked in mouse-on-mouse (MOM) Ig blocking reagent for 1 hour followed by 2.5% normal horse serum for 5 minutes. They were then probed with mouse anti-α-SMA monoclonal antibody (Sigma A2547) diluted at 1:1500 for 30 minutes. After additional blocking with MOM reagent, the tissue was stained with Vector VIP peroxidase substrate (SK-4605) for 20 seconds. Next, blocking was performed with 0.1% BSA in PBS for 1 hour. Biotinylated HABP (EMD Millipore Calbiochem) was added at 1:250 dilution in PBS and incubated overnight at 4 °C. The next day, the slides were washed in PBS and treated with Vector Elite ABC reagent (PK-7100) for 30 minutes. Next, 3,3′-diaminobenzidine (DAB) peroxidase (Vector ImmPACT) was added for 1 minute. Finally, slides were washed in PBS and stained for 1 minute with preheated methyl green (Vector H-3402) at 37 °C.

The slides were then dehydrated in ethanol and replaced in Citrasolv. Coverslips were mounted with Cytoseal-60 (Richard-Allan Scientific; San Diego, CA). Imaging acquisition and scanning was performed with an Aperio Versa light microscope (Leica; Wetzlar, Germany) with ScanScope software.

### Image signal analysis for perivascular HA content

All analyses were carried out in ImageJ using the Fiji platform build^35^. Five 40x high power fields (HPF) were analyzed in a blinded fashion for each lung. Each HPF was deconvolved along a methyl green – DAB color matrix. The DAB images were then converted to an 8-bit grayscale (0 to 255 pixel value) and used to quantitate perivascular HA signal. The outermost edge of HABP staining was outlined manually to generate a region of interest (ROI). All vessels were included per HPF to avoid selection bias. The total signal was normalized to ROI perimeter. Only vessels with sufficient circularity index ($4\pi \cdot Area/Perimete{r}^{2}$) > 0.65 were included to limit signal overestimation due to eccentricity. Vessels within close proximity to major bronchovascular structures (<200 microns) were excluded to limit contamination of HA signal by the connective tissue matrices of large airways or arteries respectively. An illustrated algorithm is provided in Supplemental File 4A. The mean alveolar background DAB staining was determined by cropping out the bronchovascular structures in each HPF and dividing the remaining signal by the remaining vessel and airway-free area. An illustrated algorithm is provided in Supplemental File 4B. A total of 5–18 vessels were analyzed per HPF, with 5 randomly selected HPFs analyzed per mouse. The vessel-to-alveolar ratio could then be calculated for each representative field. To assess the relationship between vascular HA and smooth muscle hypertrophy during hypoxia, the absolute number of HA-coated and α-SMA+ vessels in each HPF were counted by a blinded observer. To ensure that background staining did not create a bias for vessel detection rate, the number of HA-coated vessels was correlated against average HA background staining (Supplemental File 5).

### Cell culture

Primary human pulmonary artery smooth muscle cells (HPASMCs) from Lonza (Houston, TX) and ScienCell (Carlsbad, CA) were authenticated by short tandem repeat (STR) analysis, confirming 5 unique lines. Cells were cultured in complete media (SMCM) supplemented with 5% FBS, insulin, and growth factors (SMGS). Cells between passages 4 and 8 were used in all experiments. To generate superoxide, cells were exposed to hypoxanthine (HX, 0.1 mM) ± xanthine oxidase (XO, 1.6 mU/mL) for 12 hours. Hypoxia (1% O_2_ for 72 hours) was rendered with a continuous O2 controller (OKOlab; Pozzuoli, Italy). To avoid re-oxygenation, all hypoxic cell manipulations were performed in a closed modular hypoxia glovebox. To neutralize extracellular superoxide, cells were treated with bovine erythrocyte SOD1 (200 units/mL) for 12 hours, followed by further incubation in normoxia or hypoxic conditions for 72 hours. To inhibit pan-hyaluronidase activity^36^, cells were treated with high molecular weight poly-(styrene-4-sulfonate) with average mass of 1 megadalton (PSS 1 M, Sigma) at 2.5 to 10 nM. To induce enzymatic HA fragmentation, cells were treated with *S. hyaluroniticus* hyaluronidase (4 × 10^−6^ TRU) for 72 hours. To simulate extracellular changes in HA composition, cells were treated with HMWHA (1500 kD), intermediate MWHA (150 kD), and LMWHA (10 kD) at equimolar doses (LifeCore; Chaska, MN). All experiments were performed with 3–5 independent cell lines on at least 2 different days.

### HPASMC proliferation

The proliferation of HPASMCs was assessed by rate of incorporation of 5-bromo-2’-deoxyuridine (BrdU, EMD Millipore) as a reporter of DNA synthesis rate. Briefly, cells were seeded at low density (400–800 cells/well) into 96-well plates and allowed to adhere overnight. The BrdU label was added to the cells for at least 6 hours. The cells were fixed for 30 minutes with formalin and stored overnight at 4 °C. Cell labeling was detected with ELISA utilizing mouse anti-BrdU primary antibody and HRP-goat anti-mouse secondary antibody. Colorimetric peroxidase activity was detected with 3,3′,5,5′-tetramethylbenzidine (TMB) substrate, and optical density was determined at 450 nm.

### Electron paramagnetic resonance spectroscopy

To measure superoxide radical content, lung protein lysates (200–300 µg) were incubated with 200 µL of Krebs-Henseleit (KH) buffer containing diethylene-triamine-pentaacetic acid (DTPA) chelator for 20 minutes at room temperature to prevent auto-oxidation due to Fenton reaction. The 1-hydroxy-3-methoxycarbonyl-2,2,5,5-tetramethylpyrrolidine (CMH) spin probe was reacted with the lysate 1:1 ratio to completely convert unpaired oxygen species into the probe nitroxide radical (CMH-NO^•−^). The mixture was drawn up into a glass EPR capillary tube and analyzed with a Brukner EMX-Nano EPR spectrometer (Bruker; Billerica, MA). The following acquisition parameters were used: microwave frequency: 9.65 GHz; center field: 3432 G; modulation amplitude: 2.0 G; sweep width: 80 G; microwave power: 19.9 mW; number of scans: 10; sweep time: 12.11 s; and time constant: 20.48 ms. Data were expressed as concentration standardized to protein content in the sample. To determine the superoxide fraction, 100 units of bovine erythrocyte SOD1 were added to the CMH-lysate mix. The spectra obtained from EPR with and without SOD1 were aligned to each other using a least-squares fitting method. The amplitude difference of these spectra represented the SOD1-inhibitable signal, i.e. superoxide content. All data were acquired and analyzed with Brucker Xenon software.

### Statistical analysis

Data were analyzed with Prism 6 software (GraphPad; La Jolla, CA). For all mouse experiments, we used 6–10 independent animals per group, affording 80% power to exclude an effect size of at least 1.6 assuming a Gaussian distribution (α = 0.05). *In vitro* experiments were carried out with two unique cell lines on at least three separate occasions. Outliers were excluded only if they fulfilled Grubbs’ test of extreme deviation and reported in the results. We utilized parametric (two-sided t-test and 2-way ANOVA) or non-parametric (Wilcoxon signed-rank) statistical analyses as appropriate. Uniform variance was assumed for all samples and Bonferroni correction was done for multiple comparisons if applicable.

## Results

### Deficiency of SOD3 worsens hypoxic pulmonary hypertension

**Figure 1:**
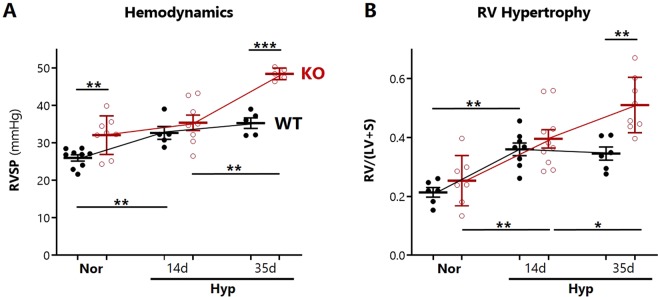
Assessment of hypoxia-induced pulmonary vascular disease. (**A**) Pulmonary hypertension developing during hypoxia in 8–10 week old male and female WT (closed black circles) and SOD3KO (KO) (red open circles) mice, determined by closed chest RV puncture. (**B**) Right ventricular hypertrophy developing during hypoxia, defined by Fulton’s index RV/(LV + S) mass ratio. Some RVSP measurements were excluded based on predefined criteria for heart rate and end-diastolic pressure. Data expressed as mean ± SEM. *p < 0.05 **p < 0.01 by 2-way ANOVA.

PH was evaluated by measurement of right ventricular systolic pressures (RVSP) and RV hypertrophy (RVH) by Fulton’s Index. In agreement with previously reported findings^29^, SOD3KO mice had higher pulmonary pressures at baseline. Our time course revealed that there were no differences between strains at an intermediate time point of hypoxia (14 days). Between 14 and 35 days of chronic hypoxia, RVSP and RVH did not worsen further in WT mice, whereas both indices continued to progress in SOD3KO mice (Fig. 1A,B). We have previously shown that inducible SMC-restricted SOD3 knockout mice (SMC-SOD3cKO) also developed worsened PH relative to their SMC-SOD3Flox controls at 35 days of hypoxia^5^.

### Deficiency of SOD3 provokes hyaluronan loss from lung tissue in hypoxia

**Figure 2:**
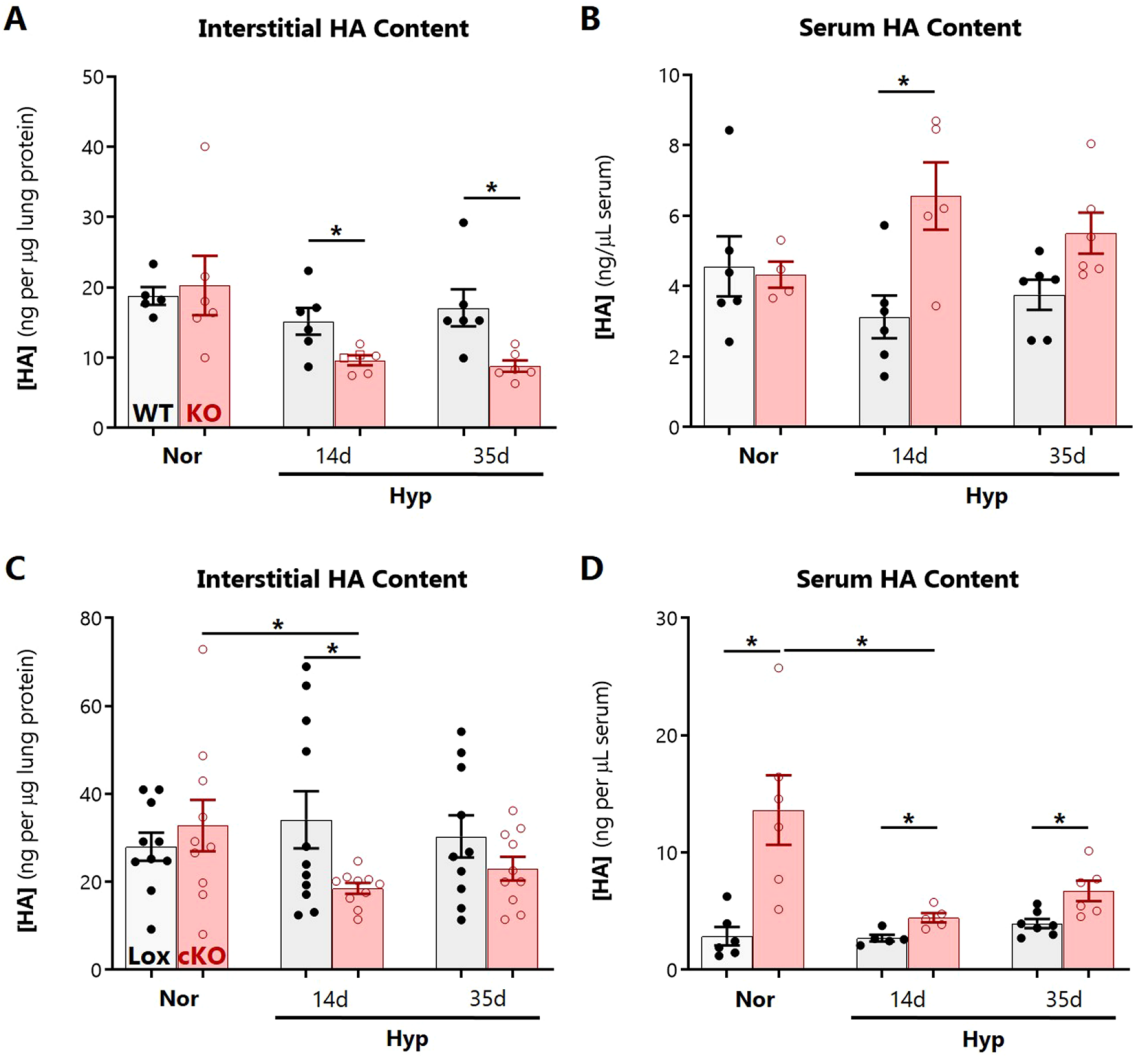
Changes in hyaluronan content during hypoxia. Changes in HA levels during hypoxia, determined by HABP-based ELISA-like assay in (**A**) lung tissue after alveolar lavage in WT (gray bars) and SOD3KO (KO, red bars) mice; (**B**) serum of WT and SOD3KO mice; (**C**) lung tissue of control SMC-SOD3Flox (Lox, gray bars) and SMC-SOD3cKO (cKO, red bars) mice after induction with tamoxifen; (**D**) serum of SMC-SOD3Flox and SMC-SOD3cKO mice. All mice were 8–10 weeks old. Males and females were equally represented in the WT and SOD3KO groups; all SMC-SOD3Flox and SMC-SOD3cKO mice were males. Data represented as mean ± SEM *p < 0.05 **p < 0.01 by 2-way ANOVA.

Because hypoxic PH severity diverged between WT and SOD3KO mice after 14 days, we tested the hypothesis that pulmonary HA would be altered by this time point. HA content was measured by an ELISA-like assay in mouse lungs that were intravascularly perfused and pre-lavaged to remove the blood and alveolar HA pools respectively. Thus, remaining HA reflects the lung interstitial and intracellular compartments. There were no significant changes in the level of lung HA in WT mice at any duration of hypoxia. In SOD3KO mice, on the other hand, tissue HA content was significantly reduced by 14 days of hypoxia, and remained decreased at day 35 (Fig. 2A). In the serum, circulating HA was significantly increased in SOD3KO mice relative to WT (Fig. 2B). We also measured HA content in lungs of SMC-SOD3cKO mice, which exhibit inducible SMC-restricted SOD3 knock-down and, similar to the total body SOD3KO mice, worsened chronic hypoxic PH^5^. In SMC-SOD3cKO mice, a similar pattern of HA tissue depletion was observed during hypoxia (Fig. 2C). Serum HA was also significantly increased in SMC-SOD3cKO in basal (normoxic) and hypoxic conditions at all time points above their floxed controls (Fig. 2D).

### Loss of SOD3 disrupts integrity of lung hyaluronan by augmenting fragmentation during hypoxia

**Figure 3:**
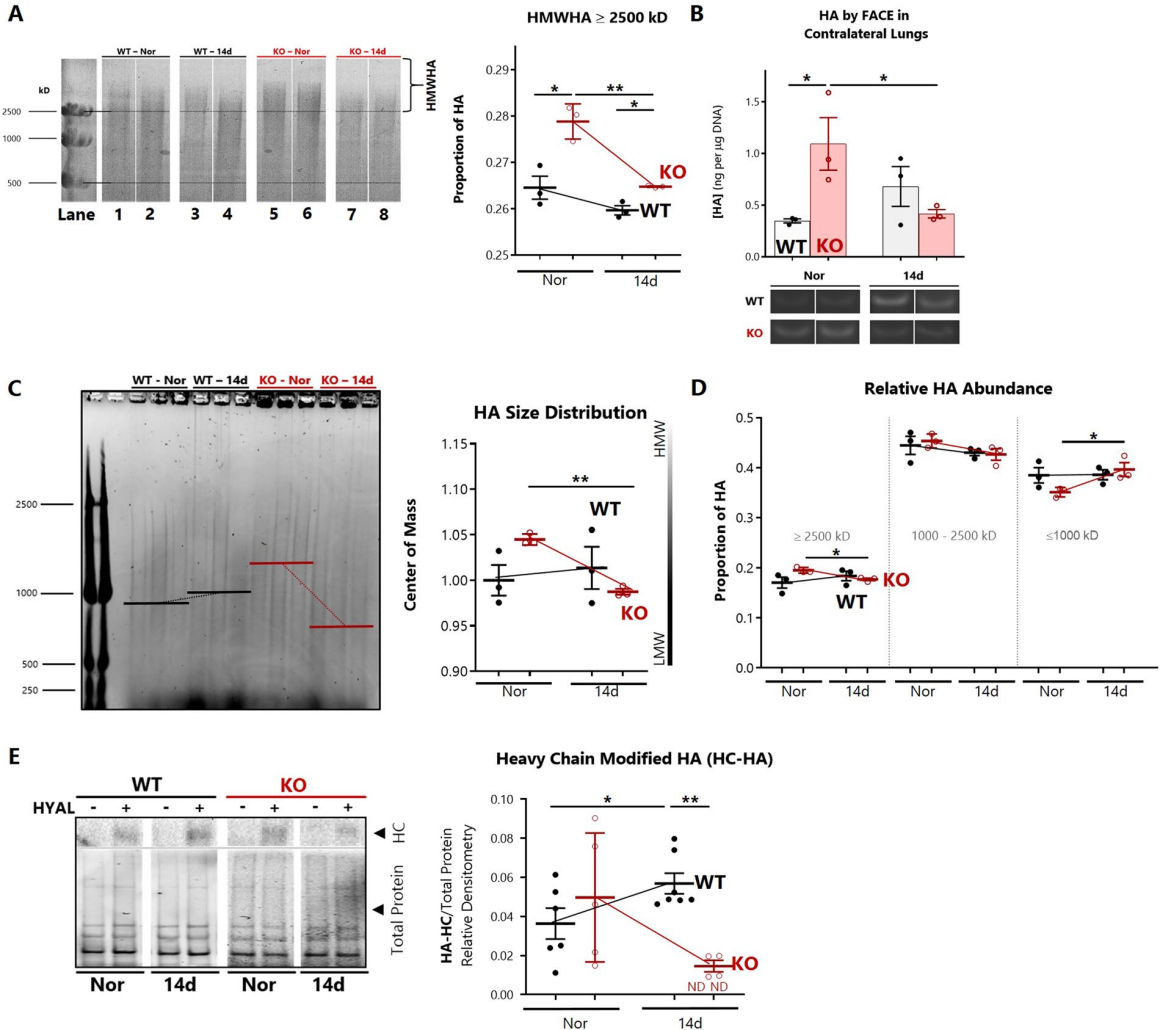
Hypoxia-induced fragmentation of hyaluronan. (**A**) Changes in high molecular weight HA (HMWHA) within lavaged lung tissue, detected by agarose gel electrophoresis of glycosaminoglycans with the corresponding quantification. Images were acquired with a standard white-light trans-illuminating scanner. All lanes were cropped from the same gel (see Supporting Information 1). Signal was verified to originate from HA by digestion with *Streptomyces hyalurolyticus* hyaluronidase. The relative amount of HMWHA (>2500 kD) was quantified and normalized to total visible HA > 500 kD to determine the proportion of >2500 kD HMWHA. Detection of intermediate and LMWHA were obfuscated by overlap with unsulfated+ chondroitin, and are therefore not visualized. (**B**) HA content by fluorophore-assisted carbohydrate electrophoresis (FACE) in the contralateral lungs with a representative blot of HA dissacharides. All regions were cropped from two gels (see Supporting Information 6A). (**C**) Electrophoresis of lung tissue HA in a separate cohort of mice, followed by gel imaging with Cy5 695/55 nm epi-fluorescence, allowing for resolution of all HA > 250 kD. (**C**) Densitometry for quantitation of HA mass distribution and (**D**) relative abundance of HA sizes. Upper and lower bounds were defined by the standard ladder. The percentage of HA in each range was determined by normalizing the size-bounded to total HA signal. (**E**) Effect of hypoxia on heavy chain (HC-HA) modification of HA in WT and SOD3KO (KO) whole lung lysates. Densitometry of HC-HA determined by probing for inter-α-inhibitor heavy chain. Signal was normalized to total protein, detected by Stain Free UV trans-illumination prior to membrane transfer (representative lanes cropped from the loading gel is shown in Supporting Information 3). Left: Representative bands showing entry of HC into the gel only upon digestion with *Streptomyces* hyaluronidase. Right: Densitometry for HC quantitation. ND = not detected. Data represented as mean ± SEM *p < 0.05 **p < 0.01 by 2-way ANOVA (n = 3–6 for each group).

Hyaluronan is susceptible to random strand scission by free radical attack, or β-1,4 glycosidic bond cleavage by endoglycosidases (hyaluronidases). To investigate whether lack of SOD3 could impact hypoxia-induced HA fragmentation, we purified glycosaminoglycans from pre-lavaged lung tissue and determined the size spectrum of HA multimers in WT and SOD3KO lungs. The characteristic wide dispersity of HA fragment sizes ranging from 250 to 2500 kD was apparent, and is demonstrated in the representative gels (Fig. 3A,C). The signal was confirmed to arise exclusively from HA by digesting samples with *Streptomyces* hyaluronidase and demonstrating abolishment of staining (Supporting Information 1A).

We calculated the relative abundance of HMWHA (≥2500 kD) in the lungs of WT and SOD3KO lungs (Fig. 3A). No changes in HA size dispersity were detected in the WT lungs at 14 days of hypoxia (lanes 1–4). In SOD3KO lungs, there was a basal enrichment of HMWHA (lanes 5 and 6). After 14 days of hypoxia, there was effacement of this HMW fraction of HA (lanes 7 and 8). To assess for fragmentation to lower molecular weights, we refined the electrophoresis protocol to resolve intermediate MW HA (≤1000 kD). In a separate cohort of mice, there were no changes in lung HA size distribution in the WT animals exposed to 14 days of hypoxia. We recapitulated the finding of selective effacement of HMWHA in SOD3KO mice, finding that the HA distribution center of mass was decreased in SOD3KO mice after hypoxia (Fig. 3C). Analysis of size distribution demonstrated that the HMWHA > 2500 kD in SOD3KO mice was converted into intermediate HA < 1000 kD after 14 days hypoxia (Fig. 3D).

Due to the potential for a size-dependent detection bias inherent to the ELISA-like method^30^ owing to nonlinear HABP binding avidity for fragments <35 kDa, we confirmed our results with fluorophore-assisted carbohydrate electrophoresis (FACE) gels (Fig. 3B). Using the contralateral lungs to those processed for HA size analysis in Fig. 3A, FACE demonstrated unchanged levels of HA in WT mice, but higher levels of HA in SOD3KO mice at baseline followed by a significant decrease at 14 days of hypoxia. Figure 3B shows the normalized concentration of lung HA disaccharides along with a representative gel. Because FACE reports the signal derived from all HA unit disaccharides, we can conclude that lung HA is both fragmented and rarified in SOD3KO mice.

HA may undergo reorganization via covalent transfer of the heavy chain (HC) from the serum protein inter-α-inhibitor (IαI). Clearance of the HC modification is a marker of increase hyaluronidase activity and HA turnover^37,38^. To determine whether loss of SOD3 alters the heavy chain modification in hypoxia, we performed immunoblotting for detection of IαI-HC, released upon hyaluronidase digestion of HC-HA complexes present in lung lysates. After 14 days of hypoxia, there was an increase in HC-HA in WT lungs and depletion of HC-HA in SOD3KO lungs (Fig. 3E).

### Increased pulmonary hyaluronan degradation in the absence of SOD3 involves enzymatic processes

**Figure 4:**
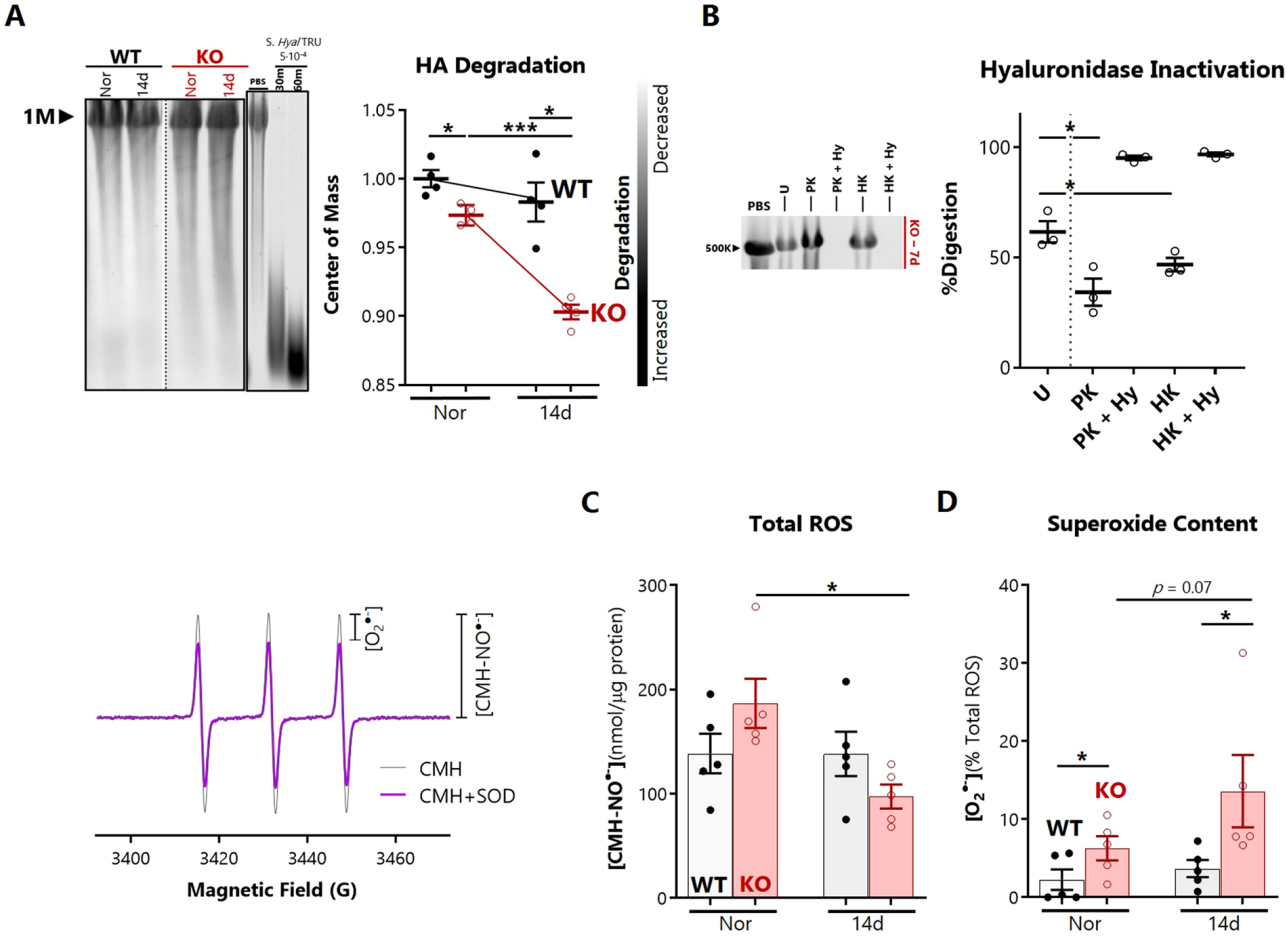
Impact of hypoxia and SOD3 on *ex vivo* enzymatic cleavage of hyaluronan. (**A**) Partial degradation of a standardized quantity of monodisperse HMWHA (1000 kD) by whole lung lysates from normoxic and hypoxic (14 day) WT and SOD3KO mice. Densitometry data are expressed as fold-change in center of mass relative to undigested PBS control. Representative lanes were cropped from the same gel (see Supporting Information 6B). (**B**) Effect of enzyme inactivation on HA degradation. Lysates were pretreated with the following conditions: U – untreated lysated; HK – heat killed; PK – proteinase K digested; Hy – addition of *Streptomyces hyaluroniticus* hyaluronidase. Densitometry data are expressed as percentage digestion (i.e. 1 - %HA remaining). (**C**) Total ROS/RNS normalized to total protein and (**D**) the specific superoxide percentage measured in frozen-and-thawed whole lung protein lysates. Left inset: representative EPR spectrum showing the total CMH-nitroxide signature and SOD1-inhibited signal respectively. Data represented as mean ± SEM *p < 0.05 **p < 0.01 by paired *t-*test (for inactivation experiments), or 2-way ANOVA.

We evaluated the HA-degrading activity in lung tissues by incubating monodisperse HMWHA (500 or 1000 kD) with lung protein lysates and then separating the resulting fragments on a 1% agarose gel. Lung lysates from 14 day hypoxia-exposed SOD3KO mice enhanced the degradation of exogenous HMWHA, observed as an increased mobility shift due to smaller-sized faster-migrating HA (Fig. 4A). Analogous results were seen as early as 7 days (data not shown). To determine whether an enzymatic mechanism of HA scission was involved, we inactivated hyaluronidases in the lung lysates by heat-denaturing or complete proteolysis with proteinase K. We found that HA degradation was significantly inhibited after these pretreatments, and restored after addition of purified *Streptomyces hyaluroniticus* hyaluronidase (Fig. 4B).

HA can be degraded by attack from multiple ROS including superoxide, hydroxyl radical, hypochlorite, and peroxynitrite^12,39^. To test whether elevated ROS, and superoxide specifically, might account for this increased HA degradation, we measured total intrinsic ROS production in thawed lung lysates by EPR spectroscopy. Next, the specific superoxide content was ascertained as the SOD-inhibitable signal detected by the EPR spin probe (CMH). Total ROS were decreased in hypoxic SOD3KO lysates (Fig. 4C) whereas superoxide was measurable and increased in SOD3KO lungs (Fig. 4D).

### Hyaluronan is preferentially lost from the perivascular matrix of small pulmonary arteries

To localize the site of HA loss, we performed immunohistochemical analyses of lung sections stained with hyaluronan-binding protein (HABP). The perivascular HABP staining signal was determined by manual segmentation of small pulmonary arteries. This signal was then normalized to the parenchymal lung staining, obtained by subtracting all bronchovascular structures. A pictorial schema of the image processing algorithm is provided (Supplemental File 4A,B).

**Figure 5:**
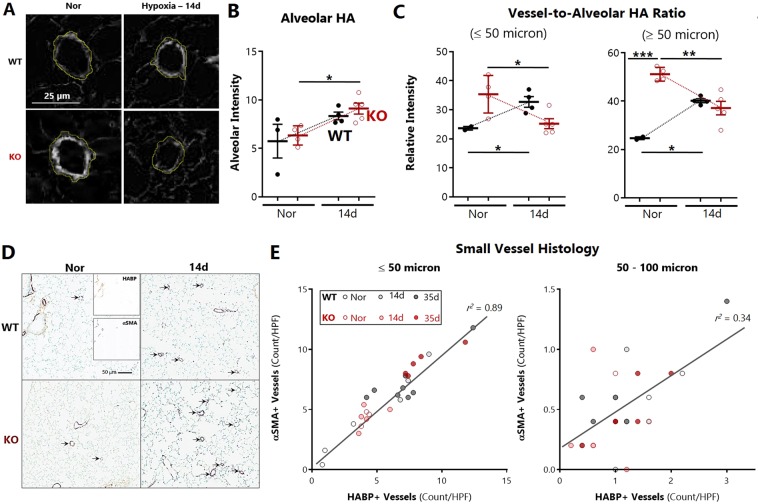
Impact of hypoxia and SOD3 on perivascular hyaluronan and arterial muscularization. Relative changes in HA in the vascular adventitial *vs*. parenchymal compartments of the lung between normoxia and 14 days of hypoxia. Quantitation of HA in HABP-stained lung sections was achieved by DAB/methyl green color vector deconvolution in ImageJ followed by signal-to-perimeter measurement of vessel edges (5–10 vessels per HPF; 5 HPF per lung) normalized to alveolar background generated by cropping away all bronchovascular structures. (**A**) Representative images of vascular HA staining after image processing. (**B**) Mean alveolar background HA staining intensity. (**C**) HA intensity expressed as vascular HA (<50 or 50–100 micron diameter) relative to the alveolar HA content in normoxic and hypoxic WT and SOD3KO (KO) mice. (**D**) Representative histo-immunologic co-staining of HABP and αSMA in lung slices. Vessels are demarcated by black arrows. (**E**) Correlation between HA-coated and de novo muscularized small vessels (≤50 microns – left panel; 50–100 microns – right panel) during hypoxic PH. Color vectors were deconvolved in ImageJ (DAB/methyl green) and independently counted in a blinded fashion. Legend: WT normoxic (open black circles), 14d hypoxic (light gray closed circles), and 35d hypoxic (darker grey closed circles); SOD3KO (open red circles), 14d hypoxic (light pink closed circles) and 35d (darker pink closed circles). 5–10 randomly selected HPF were analyzed per animal. Data represented as mean ± SEM *p < 0.05 **p < 0.01 by 2-way ANOVA.

Representative images of HA-coated vessels are shown (Fig. 5A). The parenchymal alveolar HA increased in SOD3KO mice at 14d duration of hypoxia, and did not differ between mouse strains (Fig. 5B). In small vessels (defined as <100 microns in diameter) we observed that perivascular HA was increased in SOD3KO compared to WT mice at baseline. During hypoxia, HA was progressively deposited around vessels in WT mice, with significant accumulation by 14 days. In contrast, there was a significant loss of HA from the adventitia of small vessels in the SOD3KO mice at 14 days (Fig. 5C). These results indicate that in the absence of SOD3, the outer wall of small pulmonary arteries is a major site of HA fragmentation and depletion.

Next, we tested whether vascular HA deposition co-localizes with *de novo* muscularization of pulmonary vessels during hypoxia by co-staining lung slices with HABP and α-smooth muscle actin (SMA). The images were deconvolved along the major image color vectors and vessels were independently counted in high power fields in a blinded fashion. Representative low-power fields are shown (Fig. 5D). When HA and SMA were adjudicated independently after deconvolution, we found a close correlation (r^2^ = 0.89) between the number of HA-coated (HABP+) and newly muscularized (SMA+) vessels smaller than 50 microns (Fig. 5E). When the HA-coated vessels were first identified *a priori*, they were found to be muscularized (R^2^ = 0.70 vs independent); conversely, when SMA-positive vessels were identified *a priori*, they were also found to be stained for HA (R^2^ = 0.65 vs independent). We also ensured that diminished background HA staining signal did not compromise detection of vessels; there was no correlation (R^2^ < 0.001) between signal and absolute counts (Supporting Information 5).

### SOD3 regulates the early hypoxic transcription of multiple hyaluronidase systems in the lung

**Figure 6:**
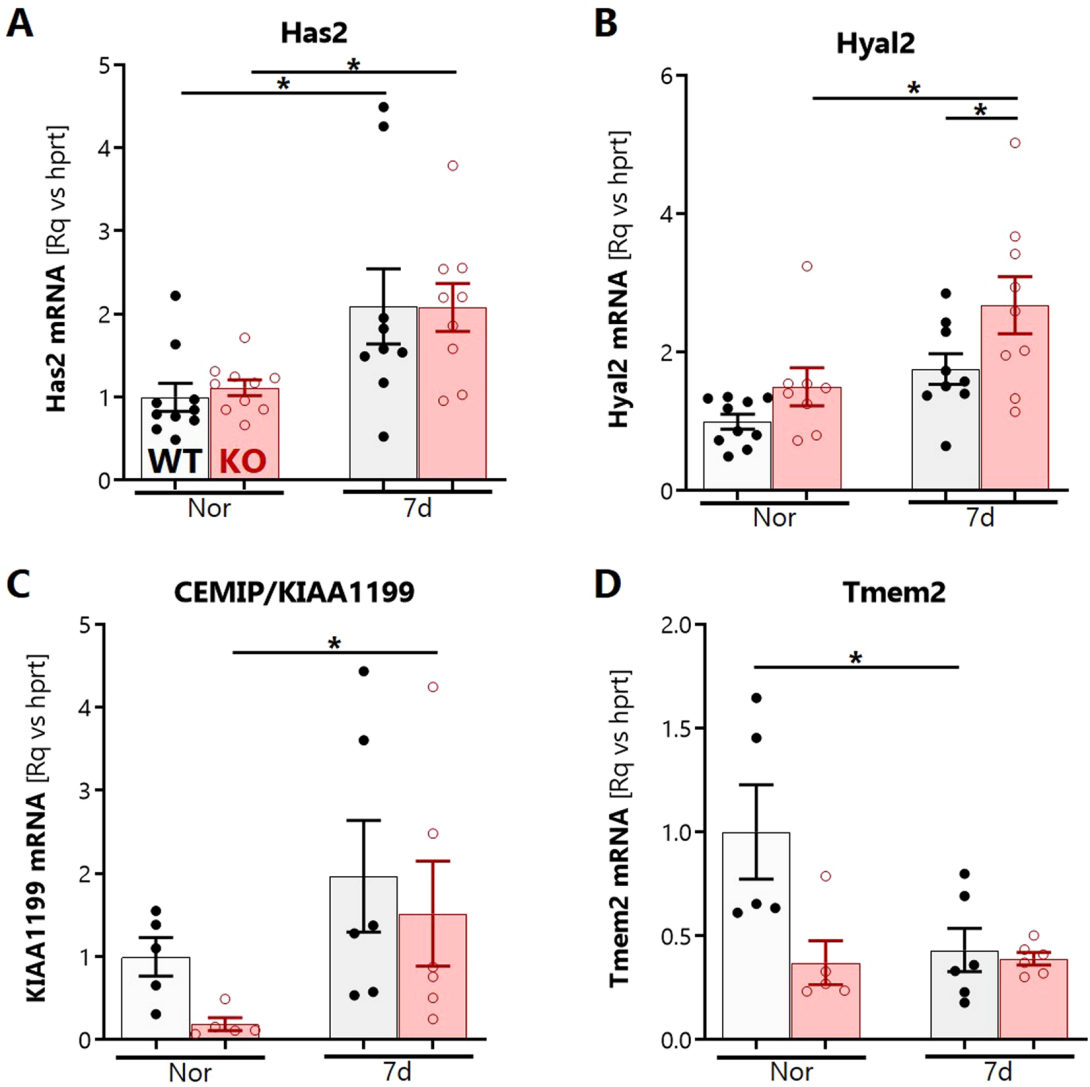
Whole lung expression of HA-regulating genes during hypoxia. Hypoxia-induced gene expression of major lung extracellular HA synthase (**A**) *has2*; and hyaluronidases (**B**) *hyal2*, (**C**) *kiaa1199/cemip*, and (**D**) *tmem2* in lavaged and finely pulverized frozen whole lung tissue of WT and SOD3KO mice. Expression was determined relative to housekeeping target *hprt* by RT-qPCR. Data represented as mean ± SEM *p < 0.05 **p < 0.01 by 2-way ANOVA.

We investigated how SOD3 influences the hypoxic expression of HA-regulating enzymes in the lung. The expression of the major hyaluronan synthase (Has2) in whole lung lysates was upregulated in hypoxia at 7 days (Fig. 6A) but did not differ between mouse strains. To further explore the contributing mechanisms to HA fragmentation, we examined the gene expression of three major HA-degrading proteins in the lung. These included Hyaluronidase-2 (HYAL2), a ROS-regulated HA lyase known to be expressed in the bronchial epithelium^40^ that has been previously implicated in idiopathic pulmonary hypertension^20,24^. *Hyal2* mRNA was transiently induced by 7 days of hypoxia, with significantly higher induction in SOD3KO mice (Fig. 6B). We then assayed expression of two CD44-independent putative hyaluronidases. The cell migration-inducing and hyaluronan-binding protein (CEMIP/KIAA1199) was expressed at significantly lower basal levels in SOD3KO mice, but was significantly induced to levels comparable to wild-type controls at 7 days of hypoxia (Fig. 6C). The mammalian homolog of zebrafish transmembrane protein-2 (TMEM2) is a recently described CD44-independent putative hyaluronidase^41^ with very high differential expression in the adult mouse lung. TMEM2 was downregulated at 7 days in WT lungs; in contrast lung TMEM2 in SOD3KO mice was expressed at lower basal levels relative to the WT strain, and expression was not influenced by hypoxia. (Fig. 6D).

### Loss of SOD3 induces the hypoxic upregulation of the hyaluronidase Hyal2 in the lung and pulmonary vasculature

**Figure 7:**
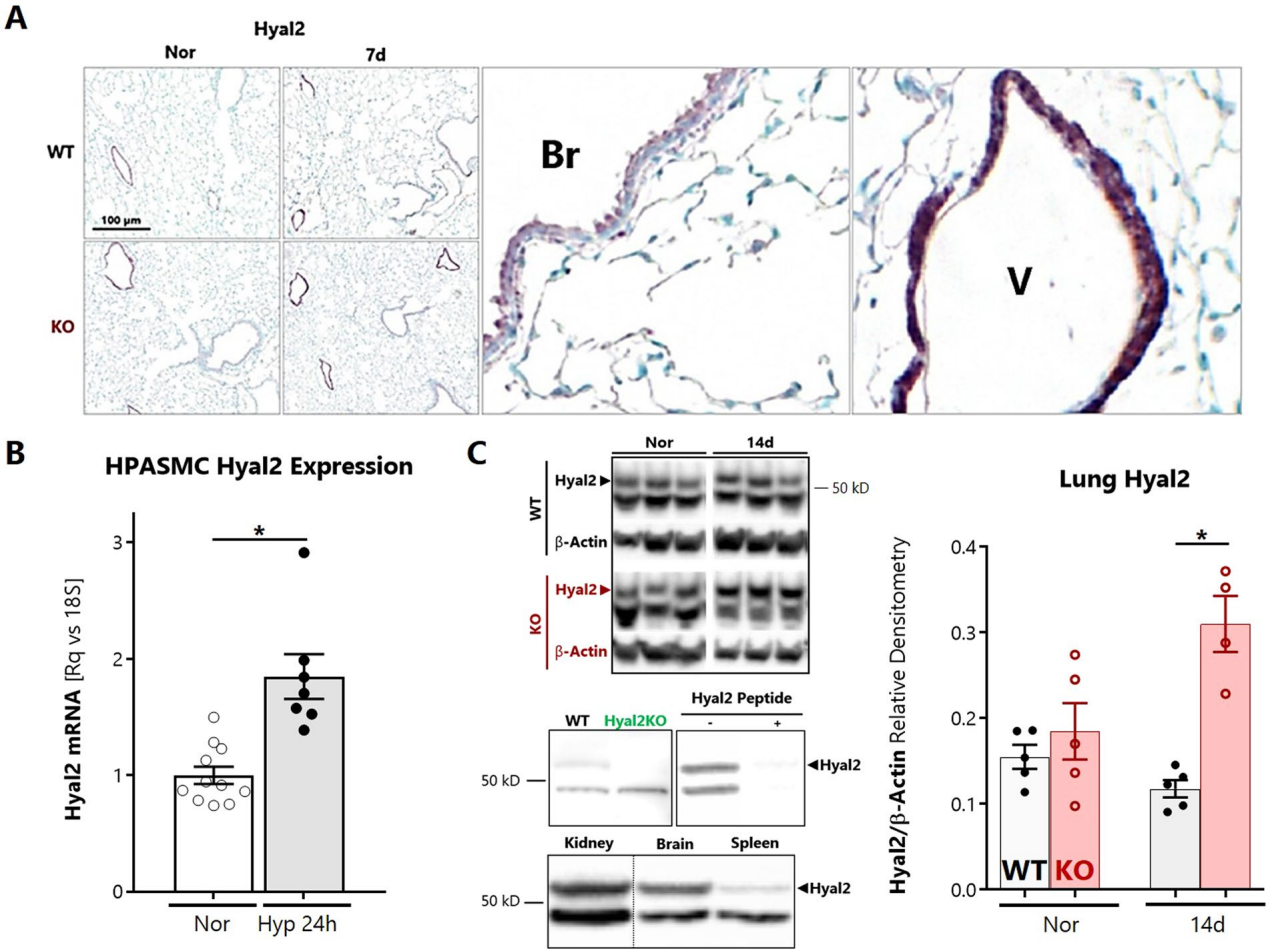
Localization and content of HYAL2 in the lung. (**A**) By immunohistology, Hyal2 was detected around bronchovascular structures. In the airways, HYAL2 is localized to the apical pole of bronchial epithelial cells (middle inset, Br – bronchus). In the vessels, HYAL2 appears to localize to the medial layer (right inset, V – vessel from same stained lung section). (**B**) Expression of Hyal2 mRNA in hypoxia-exposed HPASMCs. (**C**) HYAL2 protein expression in lung homogenates at 14 days of hypoxia. Representative regions were cropped from the same gel (see Supporting Information 6C). The lower left panel shows validation of anti-HYAL2 antibody using HYAL2KO mouse tissues, organ-specific expression in lanes from a single gel, and blocking peptide assay. Data represented as mean ± SEM *p < 0.05 **p < 0.01 by 2-way ANOVA.

To determine whether localized hyaluronidase expression would coincide with the angiocentric nature of HA degradation, we performed HYAL2 immunohistology of lung sections. As expected, HYAL2 was localized to the airway columnar epithelial layer and was concentrated at the apical surface, consistent its known GPI-dependent anchorage^40^ (Fig. 7A middle inset). In a novel observation, we found that HYAL2 was also abundantly expressed in the pulmonary arterial medial wall in normoxia and hypoxia (Fig. 7A right inset). *Hyal2* mRNA expression was increased in HPASMCs exposed to hypoxia for 24 hours (Fig. 7B). To investigate protein expression, whole lung homogenates were probed for HYAL2 by immunoblotting. HYAL2 was detected as a 54 kD-sized product; the antibody was validated by demonstrating absence of this band in Hya2KO mice^42^, decreasing expression levels in kidney, brain, and spleen respectively, paralleling the expected profile in the human protein atlas^43^, and abolition of all bands when blocked with the HYAL2 immunizing peptide. When this 54 kD band was analyzed, HYAL2 protein was induced by hypoxia, with differential upregulation evident by 14 days in the SOD3KO mouse lungs (Fig. 7C). This differential elevation of HYAL2 in SOD3KO mice was seen as early as 7 days of hypoxia (data not shown).

### Pulmonary artery smooth muscle hyaluronidase activity is SOD-regulated and directs cellular proliferation

**Figure 8:**
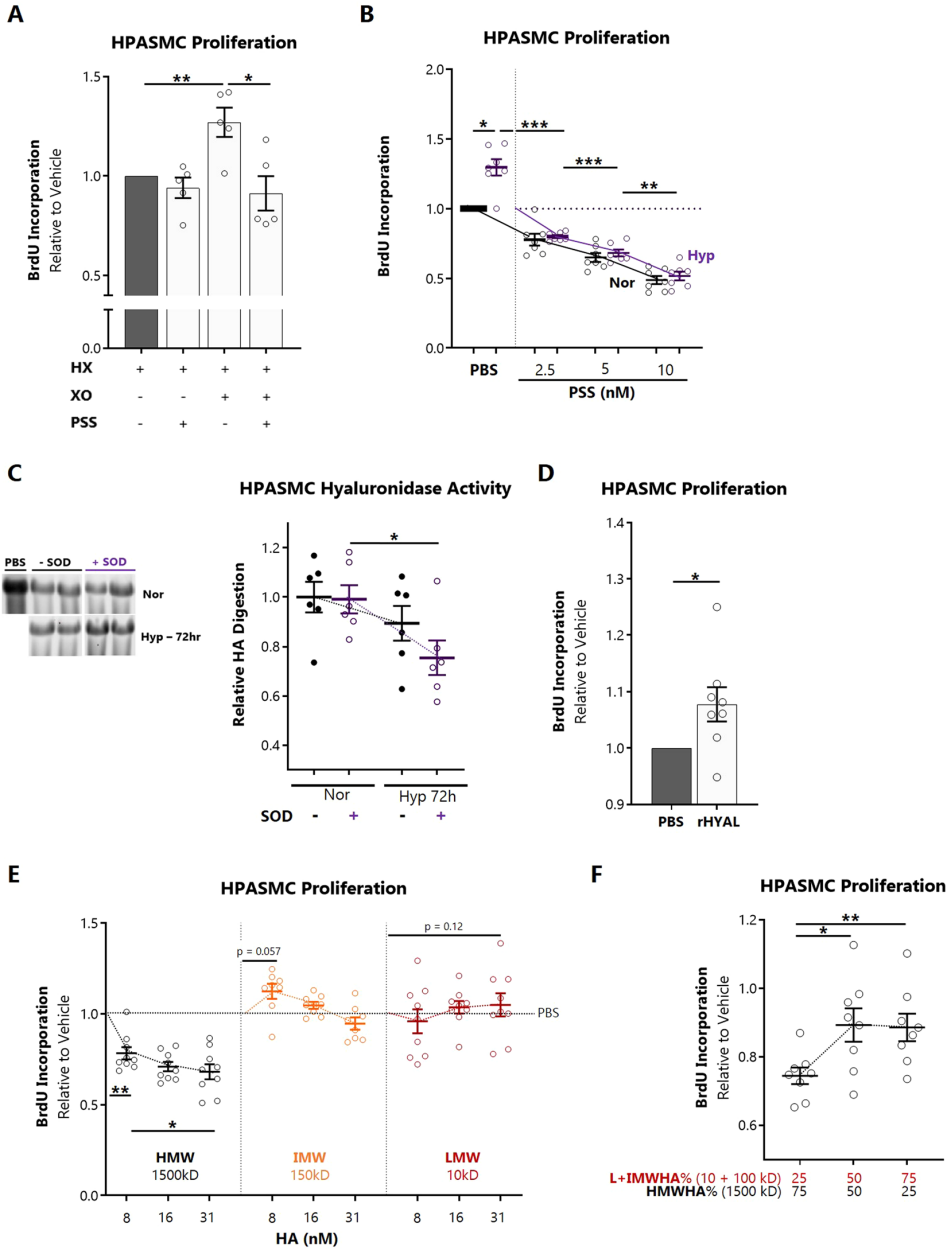
Impact of hyaluronidase activity on pulmonary artery smooth muscle cell proliferation. (**A**) Effect of a competitive pan-hyaluronidase inhibitor, poly-(4-styrenesulfonate)[PSS], on hypoxanthine/xanthine oxidase-induced HPASMC proliferation. (**B**) Dose-dependent effect of PSS on normoxic and hypoxic (1% O_2_) HPASMC proliferation. (**C**) Zymographic degradation assay for hyaluronidase activity in HPASMCs supplemented with bovine erythrocyte SOD, cultured in normoxia vs 1% O_2_ for 72 hours. (**D**) Effect of low-dose hyaluronidase (4 × 10^−6^ units) on HPASMC proliferation. (**E**) Proliferation of HPASMCs in response to three molecular weight species of HA in the high (average MW 1500 kD), intermediate (150 kD), and low (10 kD) mass ranges. (**F**) Proliferation of HPASMCs in response to replacement of growth medium with three progressive gradations (25%/50%, 50%/50%, and 75%/25%) of high vs intermediate-low molecular weight HA. All experiments were conducted with 3–5 unique primary cell lines. Data are normalized within each individual cell line and represented as mean ± SEM *p < 0.05 **p < 0.01 by 2-way ANOVA (multiple comparisons) or 2-sided paired t-test (**D**).

Proliferation of pulmonary artery smooth muscle cells (PASMCs) is a central event in the pathogenesis of vascular remodeling and pulmonary hypertension. To test whether PASMC hyaluronidase activity contributes to oxidative stress-induced proliferation, we exposed cells to the hypoxanthine (HX)/xanthine oxidase (XO) superoxide generating system and blocked hyaluronidase activity with poly(4-styrenesulfonate) (PSS). PSS is a highly efficient pan-hyaluronidase inhibitor^36^ with no endogenous antioxidant activity. PSS abrogated HX/XO-induced proliferation (Fig. 8A). PSS also dose-dependently inhibited the normoxic and hypoxic proliferation of PASMCs (Fig. 8B). To determine whether SOD could suppress hyaluronidase activity, we treated HPASMCs with 200 units of bovine erythrocyte SOD and exposed them to hypoxia. SOD treatment resulted in suppression of hyaluronidase activity only in hypoxia-exposed PASMCs, indicated by decreased reduction of the 500kD reference HA band (Fig. 8C). Exogenous hyaluronidase enzyme treatment directly increased the proliferation of PASMCs (Fig. 8D). To examine the impact of extracellular HA molecular weight composition on proliferation, cells were treated in excess with high, intermediate, or low MW HA. HMWHA inhibited proliferation, whereas intermediate and low MW species had no effect (Fig. 8E). Next, the supernatant was periodically replaced followed by re-supplementation with graded high-to-low molecular weight blends at physiologic concentrations. We observed that transition from the HWMW to intermediate and LMWHA ecosystem promoted increased proliferation (Fig. 8F).

## Discussion

Pulmonary hypertension is a complex cardiovascular disease that involves both altered redox balance and abnormal vascular extracellular matrices. Insufficient extracellular superoxide dismutase (SOD3) disrupts redox balance and contributes to PH pathogenesis. In parallel, hyaluronan (HA) is a homeostatic matrix glycan that can be damaged by oxidative stress. Therefore, the aim of our study was to elucidate how loss of the vascular antioxidant enzyme SOD3 impacts pulmonary HA fragmentation in the model of hypoxia-induced PH.

**Figure 9:**
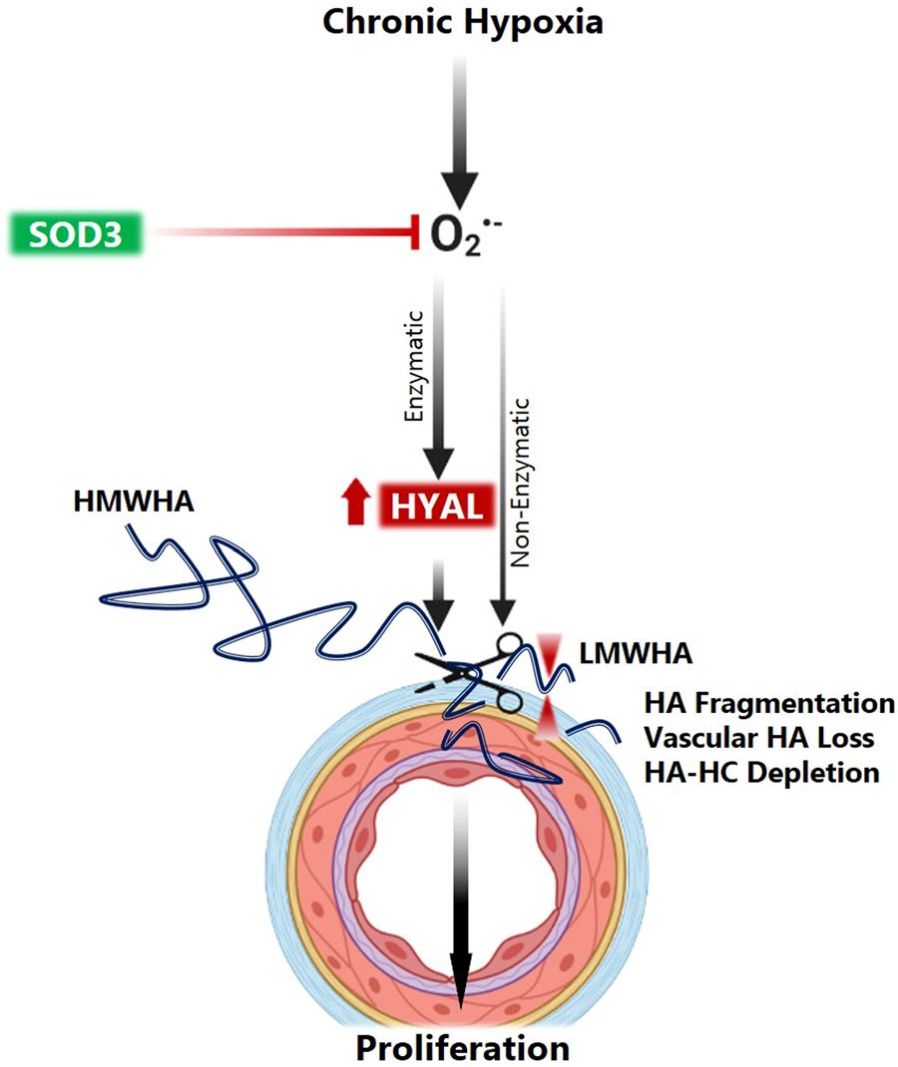
Schematic representation of major processes involved in HA remodeling in the pulmonary artery in hypoxia, exacerbated by the absence of SOD3.

Our key findings were that in the absence of SOD3, chronic hypoxia elicits major changes in HA structure characterized by: (**1**) fragmentation of HA via extracellular superoxide and hyaluronidase activity, at least partially mediated by PASMC-derived HYAL2, (**2**) spatially localized HA loss specifically around the small pulmonary vessels, (**3**) increased PASMC proliferation mediated by cleavage and loss of high molecular weight HA. Collectively, these changes indicate that extensive remodeling of the perivascular HA matrix occurs during hypoxia to instigate proliferative remodeling of the vessel wall, summarized in Fig. 9. This remodeling is promoted by loss of SOD3, and occurs at an early time point of 14 days, presaging the worsening PH severity that occurs later in SOD3 knockout mice.

Our first key finding was that sustained hypoxia induced loss of HA content, integrity and HC cross-linking, and these changes are modulated by SOD3. We observed that total tissue HA content is diminished; simultaneously, HA fragmentation into lower mass products occurs in the lungs of hypoxia-exposed SOD3KO mice. These alterations in HA content and fragmentation found in the setting of hypoxia accord with similar patterns of HA dysregulation observed in preclinical models of hypoxic PH and human PAH. For instance, early onset of HA breakdown observed in the rat monocrotaline model of PH^22^, in which lung tissue HA undergoes fragmentation and clearance 14 days, and is the resynthesized by 28 days. Acute lysis and release of basement membrane proteoglycans within hours, chiefly heparan and chondroitin sulfates, has been reported to occur during acute hypoxia exposure in rabbits^44^. In addition to lung HA depletion, we observed a concomitant increase in its serum levels in both SOD3KO and SMC-SOD3cKO mice. Differences in hypoxic trajectories between these two SOD3-deficient strains are likely driven by to the equilibrium of endovascular shedding and clearance of HA from the circulation. Elevations in circulating HA have been reported across a wide spectrum of PH subtypes including COPD-PH, chronic thromboembolic disease, and left ventricular dysfunction^45,46^. Increased circulating HA predates the phenotypic divergence of more severe PH in SOD3KO mice, raising the intriguing possibility that HA may serve as a mechanistic biomarker that can be prospectively studied in pre-symptomatic patients at risk for PH development or worsening disease status.

When considering the effects of SOD3 on HA, the enhanced fragmentation of HA observed in SOD3KO mice is consistent with previous published studies showing that SOD3 inhibits oxidant-induced HA fragmentation *in vitro*^25^, during bleomycin-induced lung injury^27^, and now extends the potential relevance of this process *in vivo* in the chronically hypoxic lung. Our study adds to these results by demonstrating that chronic hypoxia, particularly in the setting of impaired extracellular antioxidant defenses, results in the sustained loss of HA. It is also noteworthy that our group and others have reported insufficient SOD3 expression and activity in IPAH lungs^10^ and that low expression of SOD3 worsens murine PH physiology. In our study, we have also observed that deletion of SOD3 specifically in smooth muscle cells (SMC-SOD3cKO) was sufficient to elicit loss of lung HA content. Because SOD3 is predominantly synthesized by vascular SMCs and secreted in to the pulmonary artery wall, our results also reinforce the importance of SOD3 in maintaining vascular HA homeostasis. Indeed, histologic examination of the lungs revealed that HA loss is most pronounced in the pulmonary vasculature. Quantitative image analysis revealed effacement of HA from around the small pulmonary vessels (≤100 microns) in SOD3KO mice during intermediate hypoxia duration (14 days), which aligned with increased fragmentation at this time point. We cannot rule out similar changes in larger vessels (>100 microns) since these were excluded from analysis. Despite poor consensus regarding the categorization of vessels, a preponderance of data suggests that hypoxia-induced PH involves muscularization of pre-arteriolar small caliber vessels ≤100 microns^47,48^.

What are the consequences of this circumferential perivascular HA loss on vascular muscularization? Our experiments *in vitro* demonstrate that HMWHA exerts a homeostatic antiproliferative effect on HPASMCs. Loss of this normal HMWHA coating stimulates cell proliferation. Growth suppressive mechanisms of HMWHA are known to involve density-dependent Hippo pathway signaling as well as engagement of early contact inhibition through Ezrin-Radixin-Moesin (ERM) actin-binding proteins^49,50^. The buildup of perivascular HA in wildtype mice during hypoxia therefore serves to block medial proliferation. In hypoxic SOD3KO mice however, dissipation of HMWHA may therefore disinhibit proliferation, perhaps accounting for the increased muscularization and greater PH disease severity in these mice at 35 days. To our surprise, we found that SOD3KO mice displayed *elevated* levels of HMWHA basally, and yet these mice display basal (spontaneous) PH. While seemingly paradoxical in light of our discussion, this excess HMWHA may induce detrimental effects. Excess HMWHA may serve as additional substrate for fragmentation. This is supported by our finding of elevated basal hyaluronan degradation and superoxide content in SOD3KO lungs. These complexities illustrate the highly homeostatic role of HA, whereby its synthesis and catabolism must be exquisitely balanced to maintain pulmonary vascular health.

Because HA possesses broad size-dependent signaling properties, there are multiple additional mechanisms by which HA fragmentation could promote vascular remodeling. Low and intermediate weight HA are processed by PASMCs and act through RhoA kinase (ROCK) to stimulate stiffening and motility, thereby contributing to the pathologic cell phenotype^24^. Interaction of HA with the intracellular receptor for hyaluronan-mediated motility (RHAMM) directs vascular smooth muscle migration and proliferation^16^. LMWHA fragments also potentiate inflammatory responses by interstitial macrophages, and participate in co-activation of hypoxia-induced NLRP3 inflammasomes^51^. Activation of lung NLRP3 inflammasomes contributes to hypoxic PH, can be ameliorated by administration of porphyrin SOD mimetic, and involves upregulation of hyaluronidase genes^9^. Additional LMWHA-associated cellular effects include increased vascular permeability^15^ and greater invasiveness and induction of secretory phenotypes in myofibroblasts^17,52^. Perturbation of these processes are widely acknowledged in the pathogenesis of pulmonary hypertension. By maintaining HA homeostasis, SOD3 is poised to prevent these pathologic downstream sequelae.

An important consequence of increased HA fragmentation is interference with the endogenous heavy chain (HC) modification. Vascular HA matrices in the plexiform lesions of PH are often covalently modified by heavy chains transferred from serum antitrypsin inter-α-inhibitor (IαI) to HA in a reaction catalyzed by TNF-α stimulated gene 6 (TSG-6)^53^. The resultant HC-HA matrix imparts additional functional versatility to HA including a decisive role in leukocyte trafficking, tissue macrophage programming in the lung^37,54^, and regulation of fibroblast-to-myofibroblast differentiation^55^. Derangement of these processes are well-described in models of pulmonary hypertension. We found that a dramatic loss of the heavy chain-modified hyaluronan (HC-HA) occurred in the lungs of SOD3KO mice during hypoxia. Intracranial administration of oligoHA and hyaluronidase were both able to clear HC in a preterm rabbit pup model of glycerol-induced ischemic intraventricular hemorrhage^38^. In endotoxic and infectious models of acute lung injury, HC clearance coincided with induction of hyaluronidase gene expression in recruited alveolar macrophages^37^. We therefore conclude elevated hyaluronidase activity contributes to clearance of HC-HA from the lung.

Having now established that HA undergoes fragmentation, we next investigated the contribution of ROS-induced and enzyme-mediated HA cleavage. EPR of SOD3KO lungs demonstrated increased superoxide anion levels despite unchanged or lower total ROS. This later finding may reflect the action of compensatory antioxidant mechanisms known to be upregulated in SOD3KO mice^56^. Since the HA polymer has been postulated to serve as an antioxidant “sponge” for free radicals, another plausible explanation is that ROS are being scavenged in the process of HA fragmentation^57,58^. Enzyme inactivation mitigated the degradation of HMWHA by half, indicating that tissue hyaluronidases play an important role in HA cleavage, and that their activity is higher in SOD3KO lungs. Previous reports suggest that transgenic overexpression or pharmacologically delivered SOD3 attenuate hypoxic PH^7,9^. To ascertain whether external application of SOD could suppress HPASMC-induced HA fragmentation as a mechanistic explanation for its protective effect, we supplemented hypoxic cells with Cu/Zn SOD. These experiments confirmed that HPASMCs are subject to hypoxia-inducible and SOD-inhibitable hyaluronidase activity. Further studies are needed to delineate how the extracellular redox environment determines how vascular cells respond to hypoxia through HA elaboration and breakdown.

Given that enzymatic mechanisms appear to be a major contributor to hypoxic HA breakdown, we queried the gene expression of the three proteins involved in extracellular HA degradation: transmembrane protein-2 (Tmem2), cell migration-inducing and hyaluronan-binding protein (CEMIP or KIAA1199), and hyaluronidase-2 (Hyal2). HYAL2 demonstrated both hypoxic and differential induction between strains, suggesting that this enzyme shifts the equilibrium of hyaluronan turnover in favor catabolism upon challenge with hypoxia. HYAL2 is a major extracellular hyaluronidase^59^, and cleaves HMWHA into roughly 20 kD fragments to facilitate cellular internalization. In bronchial epithelial cells, HYAL2 is known to be induced by ROS in a p38MAPK-dependent manner^40^, leading to us to speculate that it could be a key hypoxia-inducible redox-regulated hyaluronidase. Indeed, we found an early increase in *Hyal2* gene expression within the lungs of SO3KO mice exposed to hypoxia (7 days) and ensuing upregulation of HYAL2 protein by 14 days. Hyal2 was highly expressed in the vascular media and in human PASMCs cultured under hypoxic conditions, induction of *Hyal2* mirrored our findings in the murine lung. Collectively, these cell studies and histologic data indicate that the pulmonary vascular smooth muscle layer is a major site of HYAL2-mediated hyaluronan catabolism during hypoxia, accounting for the perivascular epicenter of HA loss. It is also possible that this peripheral HA breakdown could be driven by other adventitial cell types. For instance, lung interstitial macrophages are recruited to the exterior vessel wall after just 4 days of hypoxia^60^ and express high levels of *Hyal2* in a model of LPS-induced lung injury^37^. HYAL2 and HA fragmentation have been implicated in matrix destruction, lung inflammation, and loss of lung function associated with COPD^61^. HYAL2 is expressed in airway epithelium and hyaluronidase activity is upregulated in asthma^62^. Our study now expands the role of HYAL2 in lung disease to hypoxia-induced PH. The published observations regarding HYAL2 expression in PH are mixed; whereas *Hyal2* gene expression in IPAH-derived SMC was lower than donor controls, its expression was higher in the lung tissues of patients with PH stemming from idiopathic pulmonary fibrosis. These discrepancies are likely due to the highly heterogeneous nature of SMC biology across pulmonary hypertension subtypes, with fundamental differences between hypoxia-induced diseases, other group 3 conditions, and idiopathic PH.

Our study has several limitations. We discovered a correlation between insufficient SOD3 and lysis of HA during the evolution of chronic hypoxia-induced PH. We also demonstrated that pharmacologic blockade of hyaluronidase activity decreased basal as well as oxidant- and hypoxia-induced PASMC proliferation. However, we have not investigated the ability of hyaluronidase inhibitors to prevent, attenuate, or rescue PH in experimental models hypoxic PH *in vivo*. Similarly, we have not examined the bioactive influence of HA fragments in other relevant pro-remodeling processes such as vascular inflammation, stiffening, and contraction. HA fragmentation-mediated downstream signaling events, and their bearing on vascular smooth muscle pathology, are well established in the literature^13,14,16,17,51,63,64^. Our results invite further evaluation of therapeutic strategies to mitigate injurious effects of HA fragmentation, which may include hyaluronidase inhibitors^65^, blockade of LMWHA-receptor signaling^63^, or restoration of SOD activity. These mechanistic and therapeutic questions are critical areas for future investigation. Secondly, we did not detect clear reductions in HA content or fragment size in wildtype mice during the course of chronic hypoxia. This implies that hypoxic HA fragmentation itself may not be a sufficient disease trigger, but that it may exacerbate or accelerate PH in susceptible backgrounds of altered extracellular redox status due to impaired SOD3 expression^10^, activity^66^, or localization^6,67^. Since our gel separation methods cannot resolve very low molecular weight and oligomeric HA species (≤50 kDa), we may have not captured the formation of these bioactive fragments in the lung tissue.

Since WT mice exhibited the contrary response of perivascular HA accumulation, it is conceivable that PH pathogenesis may proceed along divergent pathways involving either excessive HA deposition or excessive catabolism. Explanted lungs of patients nearing the end-stages of idiopathic or COPD-associated PH are marked by excessive vascular HA deposition. This may reflect the fibrotic matrix changes associated with vessel remodeling in advanced phases of disease. Cellular supernatants of isolated PASMCs from IPAH patients also demonstrate concurrently decreased hyaluronidase and HA synthase activity with net excess of HA^20^. In bleomycin-induced lung fibrosis with secondary PH, inhibition of HA synthesis with the UDP antimetabolite 4-methylumbelliferone (4-MU, hymecromone) de-remodeled hypertrophic arteries, improved oxygen transport, prevented PH onset, and reversed existing disease^24^. Therefore, successful vascular adaptation to parenchymal lung disease and/or hypoxia likely involves delicate regulation of the balance between HA breakdown and synthesis. The orchestration of HA synthase and hyaluronidase enzymes across various pulmonary vascular disease subtypes is therefore a crucial area for further study.

## Conclusion

In summary, we discovered that the extracellular redox enzyme SOD3 is required to maintain hyaluronan homeostasis in the lung and around the pulmonary vessels during hypoxia. To our knowledge, our study is the first to specifically characterize redox-sensitive hyaluronan remodeling in the lung *in vivo* as novel response to hypoxia. This remodeling occurs in the setting of deficient SOD3, and is characterized changes in HA content, polymer size, distribution, and heavy chain modification status. Pulmonary hyaluronan degradation is mainly governed by redox-sensitive hyaluronidase activity. Vessel-derived HYAL2 appears to be a key hyaluronidase involved in this process. Our findings emphasize an emerging role for the extracellular matrix in sensing and transducing oxidative stress signals derived during hypoxia. Importantly, these changes are antecedent to the onset of severe pulmonary hypertension in our mouse hypoxia model. We speculate that HA remodeling could therefore be an early step in the pathobiology of hypoxia-induced pulmonary hypertension.

## Supplementary Information

Supporting Information.
