# Supplementary material for: Extracellular Superoxide Dismutase Regulates Early Vascular Hyaluronan Remodeling in Hypoxic Pulmonary Hypertension
Source: Sci Rep. 2020 Jan 14;10:280. doi: 10.1038/s41598-019-57147-7 (PMC6959284; doi:10.1038/s41598-019-57147-7)
Supplement: Supplementary file 1 — Supporting Information. [file 41598_2019_57147_MOESM1_ESM.pdf]

SUPPORTING INFORMATION 1

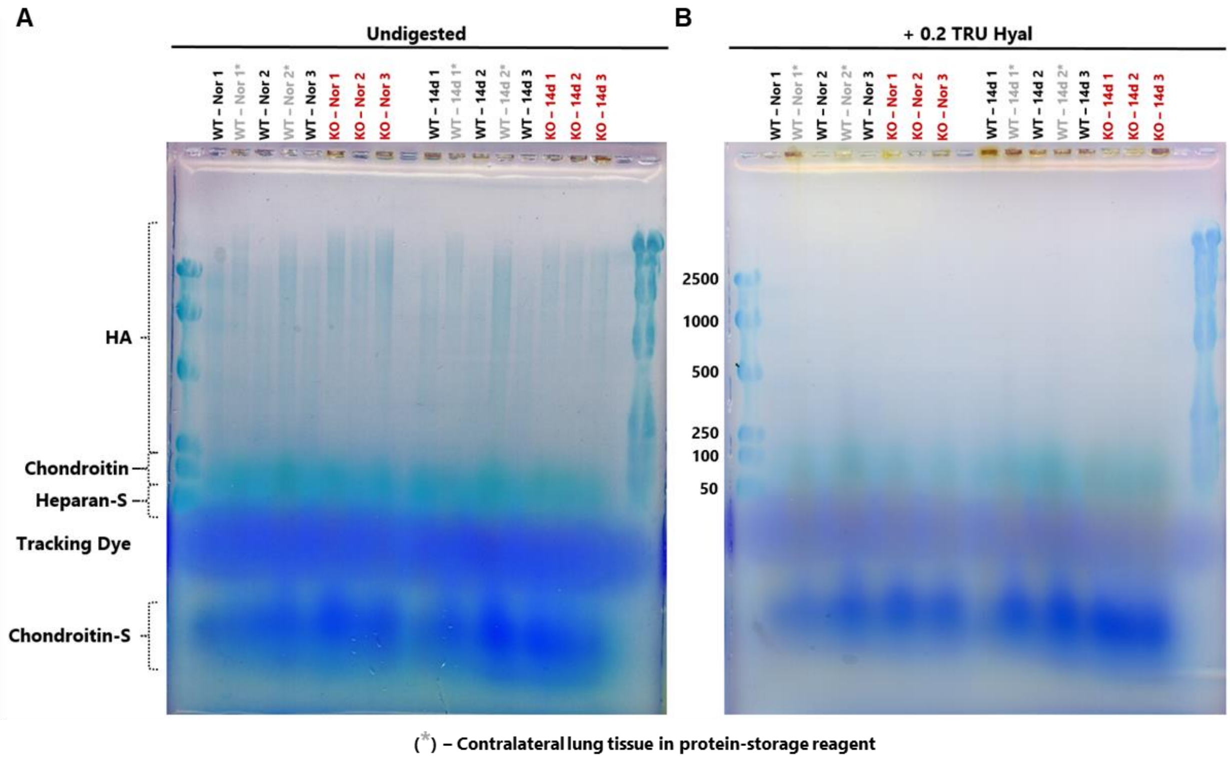

Supporting Information 1. Hyaluronan fragmentation analysis by electrophoresis. HA was isolated from lungs and separated on agarose gels and stained with Stains-All carbocyanine dye. In parallel, samples were digested with *S. hyaluroniticus* hyaluronidase prior to electrophoresis, proving that the signal is specific to HA.

SUPPORTING INFORMATION 2

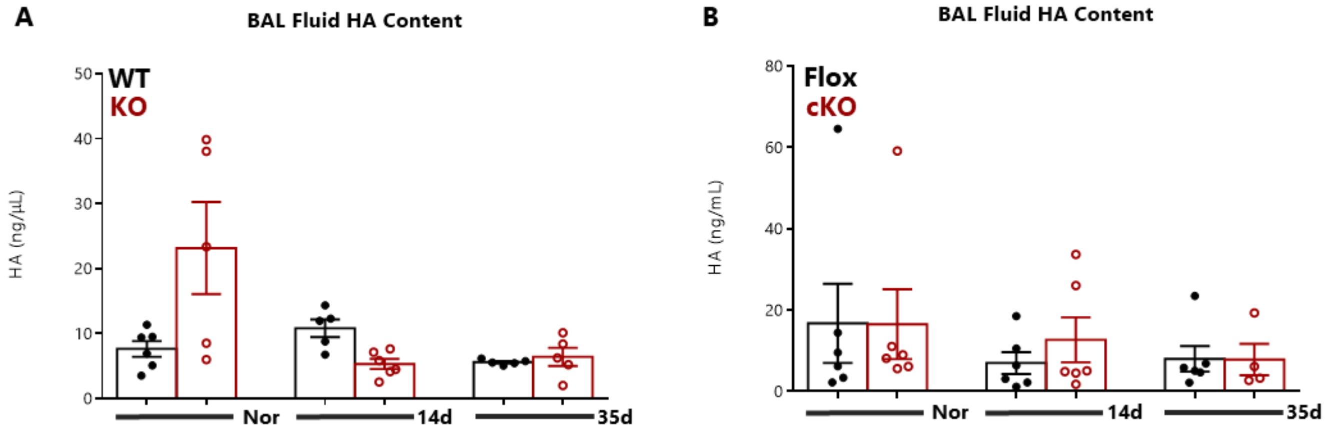

Supporting Information 2. Content of HA in the bronchoalveolar lavage fluid of (**A**) wildtype (WT, closed back circles) and SOD3KO (KO, open red circles) mice or (**B**) SOD3^{loxp} (Flox, closed back circles) and SMC-SOD3cKO (cKO, red open circles) mice.

SUPPORTING INFORMATION 3

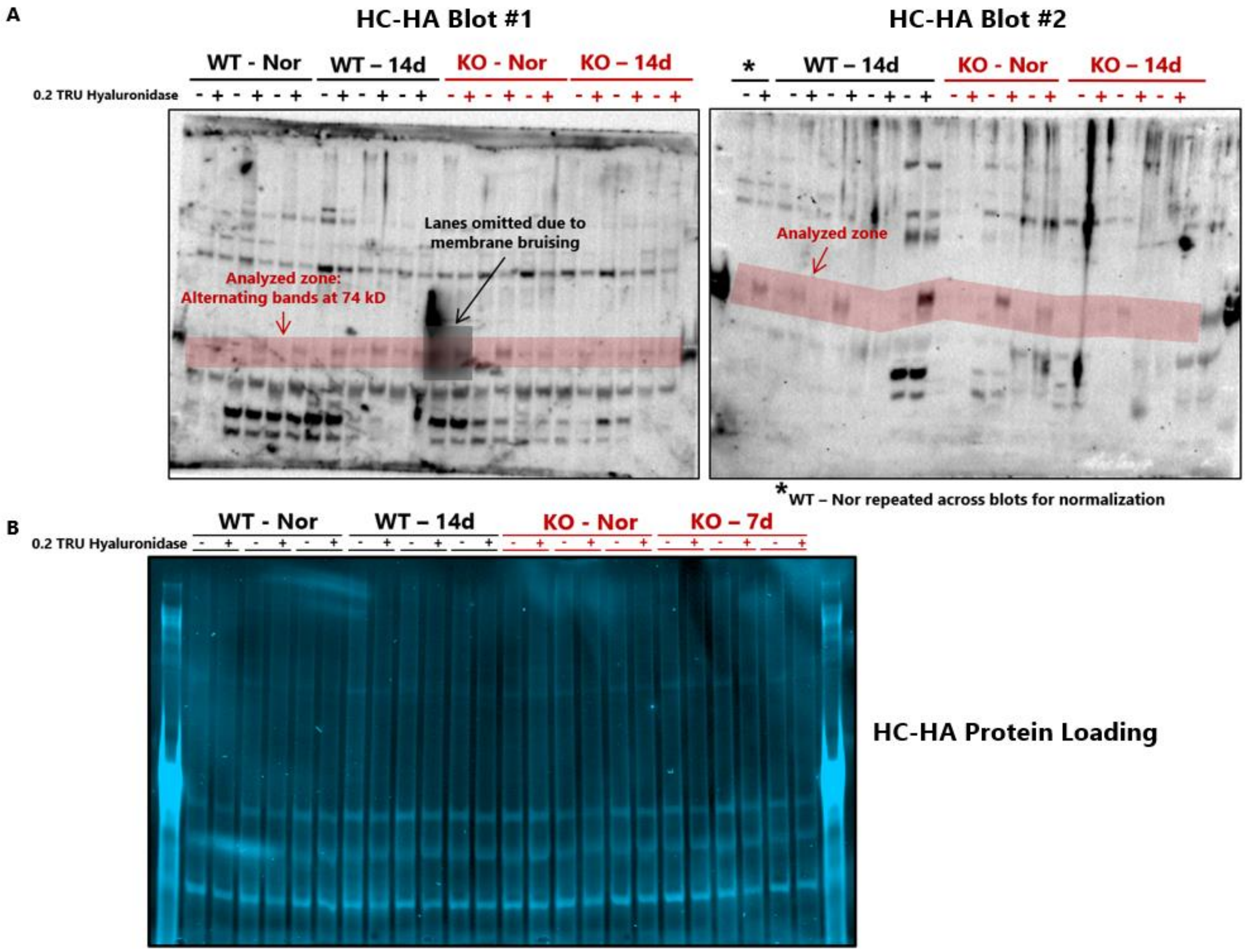

Supporting Information 3. (A) Full length membranes for detection on HC-modified HA and (**B**) representative in-gel stain-free total protein signal used for normalization.

SUPPORTING INFORMATION 4

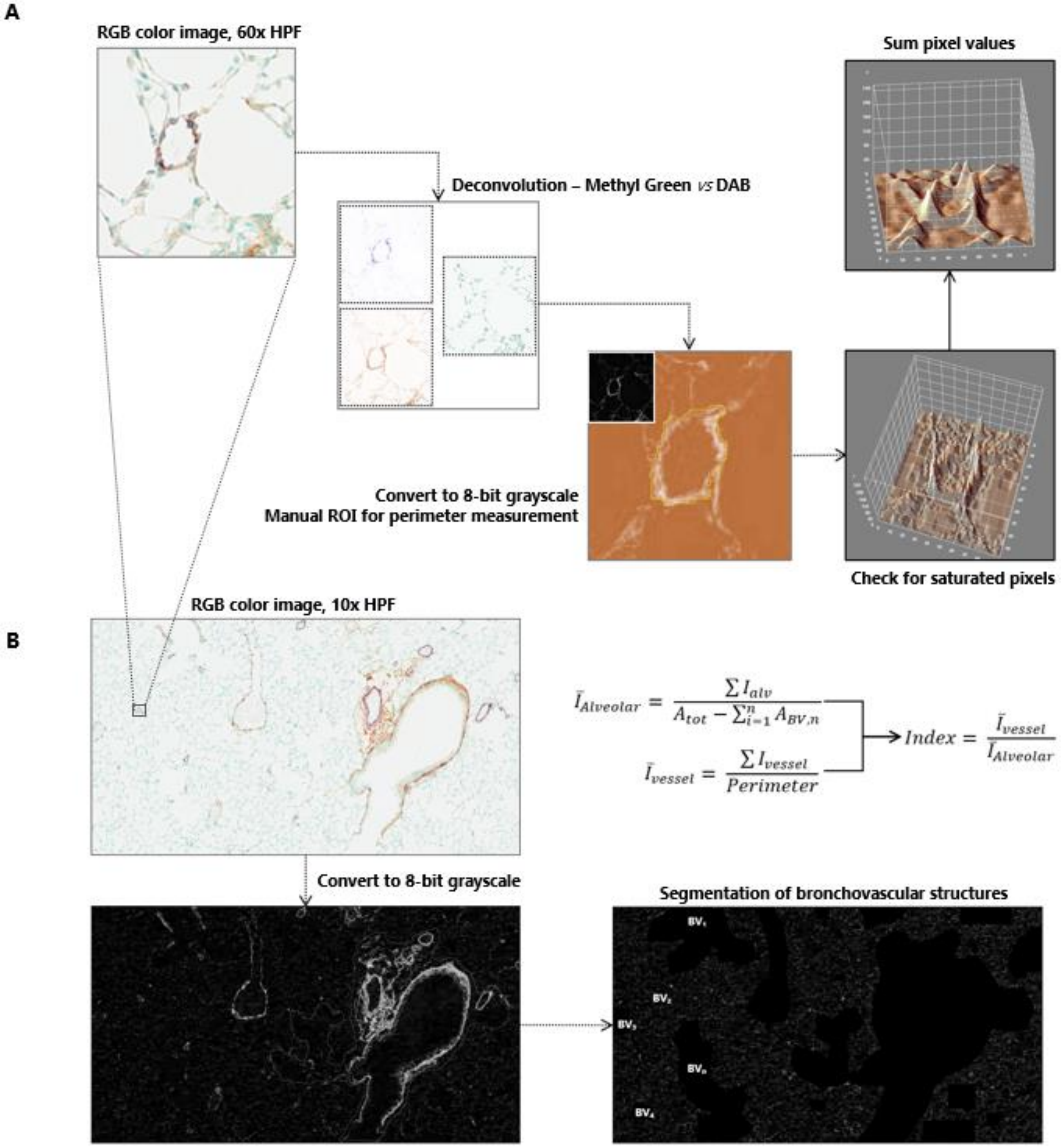

Supporting Information 4. (A) Image processing algorithm for measurement of perivascular HA pixel intensity and (**B**) background alveolar HA intensity.

SUPPORTING INFORMATION 5

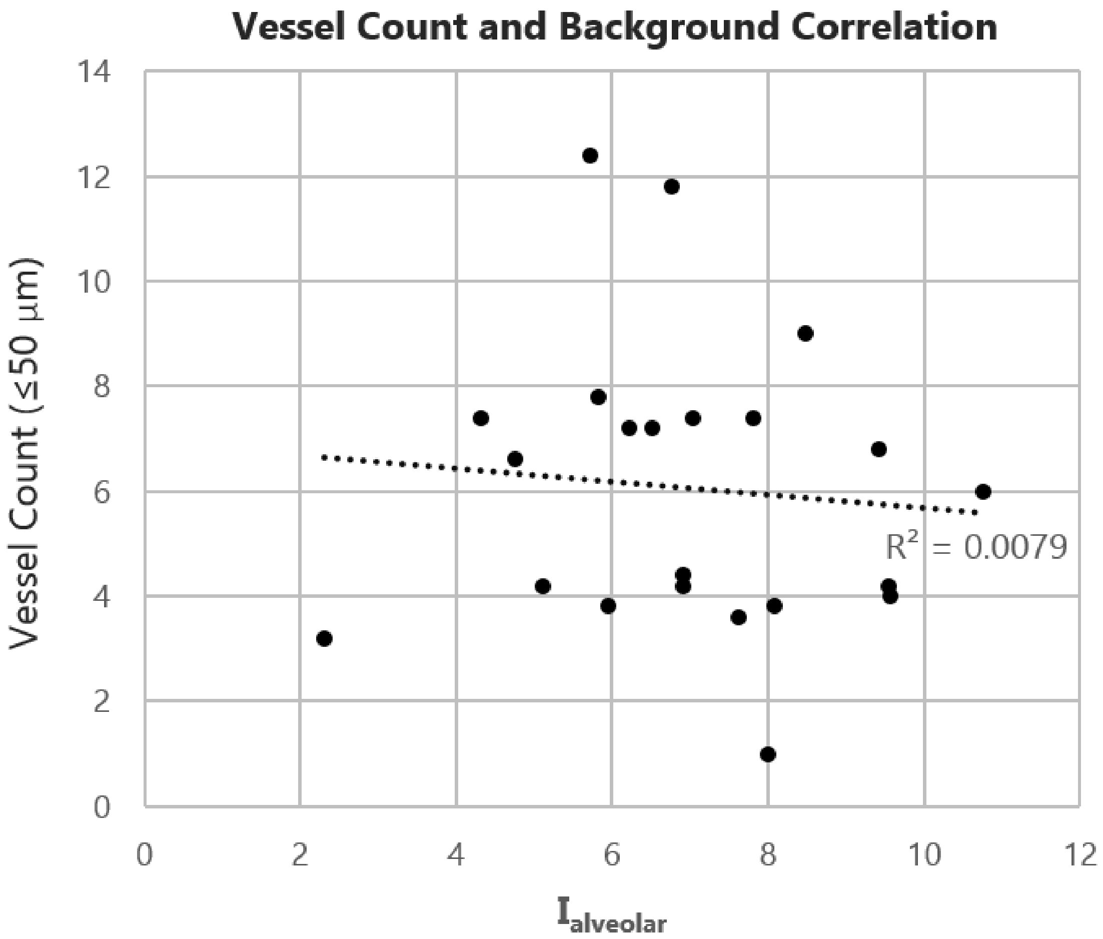

Supporting Information 5. Image processing allows for independent separation of vascular HA from background staining, as the number of HABP+ vessels is uncorrelated with background intensity.

SUPPORTING INFORMATION 6

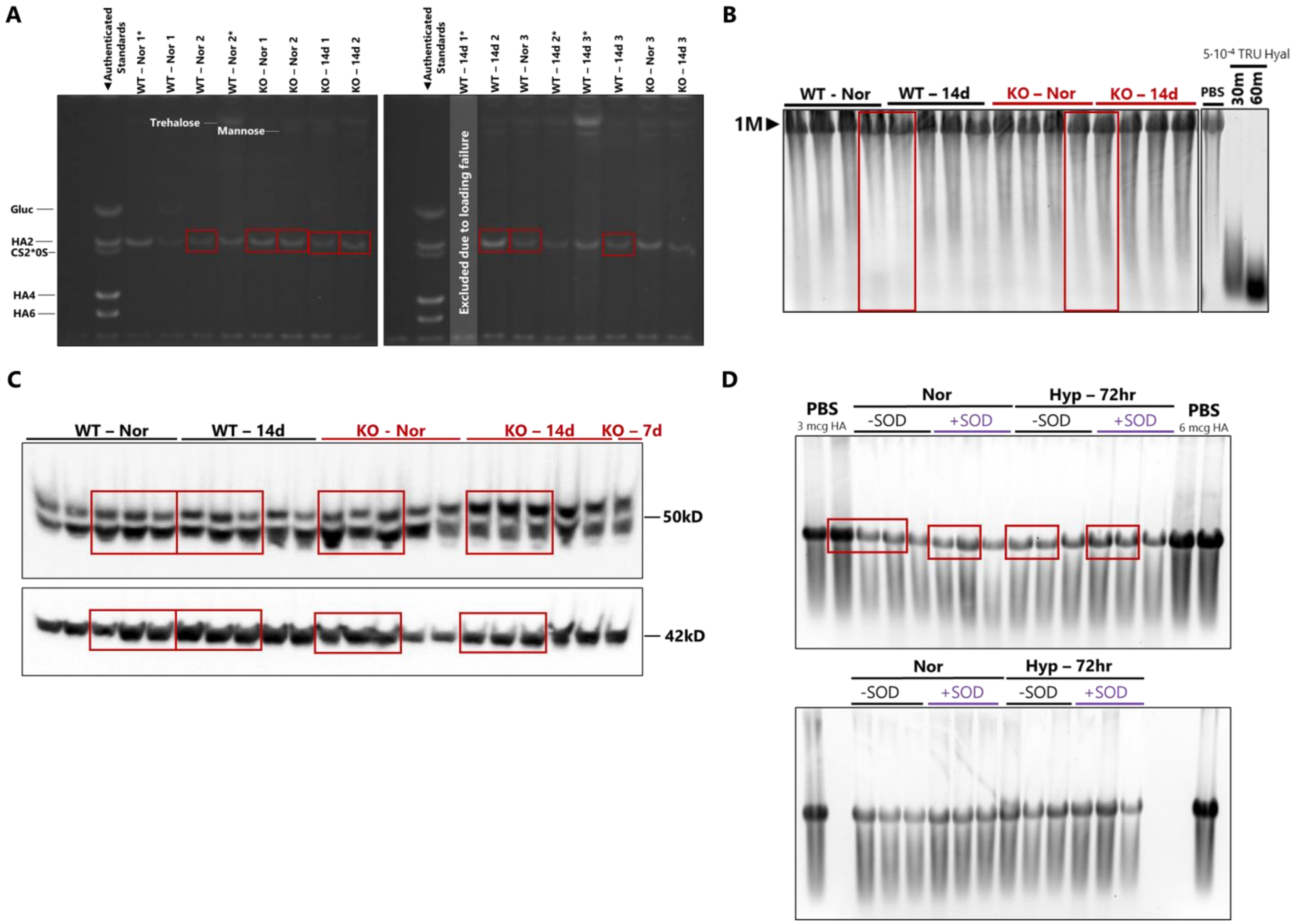

Supporting Information 6. Uncropped gels from which representative regions were selected. (**A**) Corresponding to Figure 3B. (**B**) Corresponding to Figure 4A. (**C**) Corresponding to Figure 7C. (**D**) Corresponding to Figure 8C.
